# Ruthenium atomically dispersed in carbon outperforms platinum toward hydrogen evolution in alkaline media

**DOI:** 10.1038/s41467-019-08419-3

**Published:** 2019-02-07

**Authors:** Bingzhang Lu, Lin Guo, Feng Wu, Yi Peng, Jia En Lu, Tyler J. Smart, Nan Wang, Y. Zou Finfrock, David Morris, Peng Zhang, Ning Li, Peng Gao, Yuan Ping, Shaowei Chen

**Affiliations:** 1 Department of Chemistry and Biochemistry, University of California, 1156 High Street, Santa Cruz, CA 95064 USA; 2 Key Laboratory of Photochemical Conversion and Optoelectronic Materials, Technical Institute of Physics and Chemistry, Chinese Academy of Sciences, 100190 Beijing, China; 3 Department of Physics, University of California, 1156 High Street, Santa Cruz, CA 95064 USA; 4 New Energy Research Institute, School of Environment and Energy, South China University of Technology, Guangzhou Higher Education Mega Center, 510006 Guangzhou, Guangdong China; 5 Science Division, Canadian Light Source Inc., 44 Innovation Boulevard, Saskatoon, SK S7N 2V3 Canada; 6 CLS@APS, Sector 20, Advanced Photon Source, Argonne National Laboratory, 9700 Cass Avenue, Lemont, IL 60439 USA; 7 Department of Chemistry, Dalhousie University, 6274 Coburg Road, Halifax, NS B3H 4R2 Canada; 8 International Center for Quantum Materials, Peking University, 100871 Beijing, China; 9 Electron Microscopy Laboratory, School of Physics, Peking University, 100871 Beijing, China; 10 Collaborative Innovation Centre of Quantum Matter, 100871 Beijing, China

## Abstract

Hydrogen evolution reaction is an important process in electrochemical energy technologies. Herein, ruthenium and nitrogen codoped carbon nanowires are prepared as effective hydrogen evolution catalysts. The catalytic performance is markedly better than that of commercial platinum catalyst, with an overpotential of only −12 mV to reach the current density of 10 mV cm^-2^ in 1 M KOH and −47 mV in 0.1 M KOH. Comparisons with control experiments suggest that the remarkable activity is mainly ascribed to individual ruthenium atoms embedded within the carbon matrix, with minimal contributions from ruthenium nanoparticles. Consistent results are obtained in first-principles calculations, where RuC_x_N_y_ moieties are found to show a much lower hydrogen binding energy than ruthenium nanoparticles, and a lower kinetic barrier for water dissociation than platinum. Among these, RuC_2_N_2_ stands out as the most active catalytic center, where both ruthenium and adjacent carbon atoms are the possible active sites.

## Abstract (web summary)

Electrochemical water splitting is promising for sustainable energy, but relies on development of electrocatalysts for hydrogen evolution. Here the authors report nitrogen- and ruthenium-codoped carbon nanowires in which ruthenium atoms in a carbon matrix drive electrocatalysis of hydrogen evolution

## Introduction

Hydrogen evolution reaction (HER) plays a significant role in electrochemical water splitting for clean and sustainable hydrogen energy^1,2^. In practice, room-temperature water electrolysis can be performed in both acidic and alkaline electrolytes, where platinum-based nanoparticles generally serve as the catalysts of choice. Whereas numerous studies have been carried out in acid, the high cost of proton exchange membranes^3^ as well as the sluggish electron-transfer kinetics of oxygen evolution reaction in acidic media^4^ have greatly hampered the widespread applications of acidic water electrolyzers. Such issues can be mitigated when the reactions are carried out in alkaline media, where HER involves three key steps^5,6^,

1 $${\mathrm{H}}_{\mathrm{2}}{\mathrm{O + }} \ast {\mathrm{ + e}}^{\mathrm{ - }} \to {\mathrm{H}} \ast {\mathrm{ + OH}}^{\mathrm{ - }}\left( {{\mathrm{Volmer}}} \right)$$

2 $${\mathrm{H}} \ast {\mathrm{ + H}}_{\mathrm{2}}{\mathrm{O + e}}^{\mathrm{ - }} \to {\mathrm{H}}_{\mathrm{2}}{\mathrm{ + OH}}^{\mathrm{ - }}\left( {{\mathrm{Heyrovsky}}} \right)$$

3 $${\mathrm{2H}} \ast \to {\mathrm{H}}_{\mathrm{2}}\left( {{\mathrm{Tafel}}} \right)$$

with * being the active site. The HER performance is generally discussed within the context of the Volmer–Heyrovsky or Volmer–Tafel pathway. Interestingly, whereas alkaline HER entails water dissociation, a unique step that is unseen in acid HER, the adsorption free energy of H to the catalyst surface (Δ*G*_H*_) remains an effective descriptor^7–11^. In conjunction with the calculations of the energy barrier of water dissociation^12^, the active sites as well as the reaction pathways can be resolved.

Nevertheless, alkaline water electrolysis come with a significant disadvantage of its own, which is the markedly diminished HER electron-transfer kinetics catalyzed by platinum (about two orders of magnitude lower than that in acid)^13^. Thus it is of both fundamental and technological significance to improve the performance of Pt-based HER catalysts^6^ or develop viable alternatives that are of low cost and high performance for HER electrocatalysis in alkaline electrolytes (e.g., transition metal oxides, chalcogenides, and phosphides)^14–17^. Toward this end, non-platinum noble metals, such as ruthenium, rhodium, palladium, and iridium^18–21^, have also been attracting particular attention because of their apparent performances. Of these, ruthenium, in the form of nanoparticles, alloys, and oxides, has been found to display an HER activity that is comparable to that of commercial platinum catalysts in alkaline solutions^22–29^. For instance, Mahmood et al.^18^ reduced RuCl_3_ onto nitrogen-doped two-dimensional (2D) frameworks by NaBH_4_, and the composite was then pyrolyzed producing ruthenium nanoparticles of 1.6 nm in diameter dispersed in the carbon matrix. The sample exhibited an apparent HER activity with an overpotential (*η*_10_) of only −25 mV to reach the current density of 10 mA cm^−2^ in 1 M KOH. In another study^30^, Zheng et al. prepared a composite with ruthenium nanoparticles (dia. 2 nm, consisting of a mixture of face-centered cubic (*fcc*) and hexagonal close-packed (*hcp*) ruthenium) and carbon nitride (C_3_N_4_) by annealing the product at elevated temperatures. The catalyst showed an *η*_10_ of −79 mV in 0.1 M KOH. In these studies, the HER activity was generally ascribed to ruthenium nanoparticles^18,30,31^. In a more recent study^32^, Zhang et al. immersed a polyaniline-coated graphite foam into a Ru^3+^ solution, and after pyrolysis at controlled temperatures, transmission electron microscopic (TEM) measurements showed that Ru nanoparticles (2.1–8.3 nm in diameter) and atomic species were formed and dispersed on the carbon surface. The resulting sample showed an *η*_10_ of −21 mV in 1 M KOH. Based on results of a poisoning experiment with KSCN, the activity was attributed to atomically dispersed Ru in the nitrogen-doped carbon matrix instead of the Ru nanoparticles; yet the detailed structures of the atomic species and hence the catalytic active centers remained unresolved. In a series of other studies^27,28,33^, ruthenium ions were embedded into the C_3_N_4_ skeleton by complexation to the pyridinic N moieties of the tri-s-triazine units, forming a structure with isolated Ru metal centers; yet the corresponding HER activity, while remarkable, remained subpar as compared to that of Pt/C. This raises an interesting question: will ruthenium single atom catalysts ever rival commercial Pt/C in HER electrocatalysis? This is the primary motivation of the present study.

Herein, we prepared a nanocomposite based on ruthenium and nitrogen codoped carbons and examined the impacts of both ruthenium nanoparticles and atomic ruthenium sites on the HER performance. Experimentally, a melamine–formaldehyde (MF) polymer was coated onto tellurium nanowires (Te NWs), and the core-sheath NWs were pyrolyzed at controlled temperature after the addition of a calculated amount of ruthenium(III) chloride, leading to the formation of Ru,N-codoped carbon NWs where both ruthenium nanoparticles and ruthenium single atoms were embedded within the carbon matrix. Notably, the sample exhibited a remarkable HER activity in alkaline media, a performance even significantly better than that of commercial Pt/C. Control experiments showed that the HER activity was primarily due to atomically dispersed Ru coordinated to N and C, with minor contributions from ruthenium nanoparticles. Consistent results were obtained in computational studies based on first principles calculations, where the HER active sites were most likely the ruthenium atomic centers involved in (undersaturated) coordination with N and C (RuC_*x*_N_*y*_). Carbon atoms adjacent to the Ru center were also possible active sites based on favorable H-binding energies and relatively low formation energies. The corresponding energy barriers for water dissociation (Volmer reaction) were then evaluated by using the climbing-image nudged elastic band (CI-NEB) method^12,34^, which were found to be even lower than that on Pt^35^, indicating a faster kinetic process.

## Result

### Synthesis and characterization

**Fig. 1:**
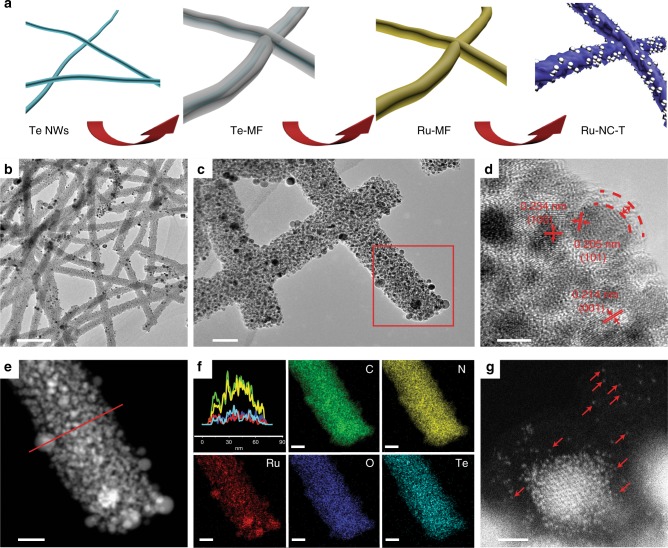
Schematic of sample preparation and transmission electron microscopic (TEM) studies. **a** Synthetic procedure of the Ru-NC-*T* samples. Te NWs denotes tellurium nanowires, Te@MF refers to tellurium nanowires with a melamine–formaldehyde resin shell, Ru-MF indicates the incorporation of ruthenium precursors into Te@MF, and Ru-NC-*T* signifies Ru,N-codoped carbon prepared by pyrolysis of the Ru-MF nanowires at different temperatures. **b**–**d** Representative TEM images of Ru-NC-800 at different magnifications. Scale bars are **b** 500 nm, **c** 50 nm, and **d** 5 nm. **e** High-angle annular dark-field scanning TEM image of the red area of **c**. The scale bar is 20 nm. **f** Cross-sectional elemental distributions by line scans along the red line in **e**. The colors of the elemental maps of C, N, O, Te, and Ru correspond to those in the line scan spectra. Scale bars are all 20 nm. **g** A zoom-in of **e**, where red arrows signify ruthenium single atoms. The scale bar is 1 nm

One-dimensional Ru,N-codoped carbon NWs were synthesized by using Te NWs as sacrificial templates^36–39^. The procedure includes four major steps (Fig. 1a): (i) hydrothermal synthesis of Te NWs, (ii) formation of a MF resin shell on Te NWs (Te@MF), (iii) reaction of Te with RuCl_3_ to incorporate Ru precursors into Te@MF (Ru-MF); and (iv) pyrolysis of the Ru-MF NWs at elevated temperatures to produce Ru,N-codoped carbon nanofibers (Ru-NC-*T*, with *T* being the pyrolysis temperature). In this procedure (details in the Methods section), melamine serves as a carbon and nitrogen source and formaldehyde as a second carbon source and linking agent.

When RuCl_3_ was added into the Te NW dispersion, the solution color was found to change from blue to dark brown, indicative of reaction between Te and Ru^3+^ forming RuTe_*x*_ complexes. From the TEM image in Supplementary Figure 1a, one can see that the resulting NWs are 80–100 nm in diameter and several microns in length, and the surface is decorated with a number of dark spots, which are most likely the RuTe_*x*_ complexes. This can be better observed at higher magnifications (Supplementary Figure 1b). Yet, no well-defined lattice fringes can be seen (Supplementary Figure 1c), suggesting amorphous characteristics of the resin and the complexes. Elemental mapping analysis shows that the elements of C, N, O, Te, and Ru are distributed rather evenly throughout the NWs (Supplementary Figure 1d-f). After pyrolysis at elevated temperatures, dark-contrast objects were formed and embedded within the carbon matrix (Fig. 1b–d and Supplementary Figure 2), which exhibited well-defined lattice fringes (Fig. 1d), with interplanar spacings of 0.234, 0.214, and 0.205 nm that are characteristic of the (100), (001), and (101) planes of *hcp* Ru (JCPDS-ICDD card No. 06-0663). This indicates the formation of Ru nanoparticles, which were encapsulated within a thin carbon layer of 2–3 nm (Fig. 1d). The ruthenium nanoparticles showed a marked increase of the average diameter with increasing pyrolysis temperature, ca. 2.0 nm for Ru-NC-500, 2.5 nm for Ru-NC-600, 3.7 nm for Ru-NC-700, and 4.7 nm for Ru-NC-800 (Supplementary Figure 2). The formation of ruthenium nanoparticles is likely due to reduction of the RuTe_*x*_ complex in Ru-MF by carbon at high temperatures, which also facilitated the migration and Ostwald ripening of the nanoparticles, leading to an increase of the nanoparticle size. Furthermore, one can see that the NW morphologies did not change appreciably after pyrolysis, though with a somewhat smaller cross-sectional diameter (ca. 60 nm) than that of Ru-MF, likely due to partial decomposition of the MF resin during the carbonization process (Fig. 1b–d and Supplementary Figure 2). Elemental mapping based on energy-dispersive X-ray analysis shows that all elements of C, N, O, Te, and Ru remained readily visible (Fig. 1e, f); and whereas the distributions of C, N, O and Te were rather uniform (no other element was detected, Supplementary Figure 3), ruthenium exhibited apparent clustering, coincident with the formation of ruthenium nanoparticles. Remarkably, in addition to Ru nanoparticles, a number of Ru single atoms can also be readily identified within the carbon matrix, as manifested in double aberration-corrected high-angle annular dark-field scanning TEM (HAADF-STEM) measurements and highlighted by red arrows in Fig. 1g and Supplementary Figure 4.

**Fig. 2:**
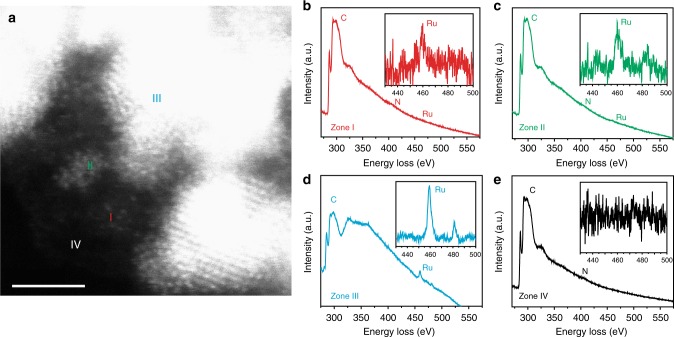
Transmission electron microscopic and electron energy loss spectroscopic measurements. **a** Representative high-angle annular dark-field scanning TEM image of Ru-NC-700. The scale bar is 2 nm; **b**–**e** electron energy loss spectroscopic spectra of zones (I)–(IV) in **a** that correspond to ruthenium single atoms, ruthenium nanoclusters, ruthenium nanoparticles, and ruthenium-free carbon matrix, respectively. The insets are the respective zoom-in within the energy range of 430–500 eV

To further confirm the formation of Ru single atoms and to examine the structure of the various forms of ruthenium in the carbon matrix, electron energy loss spectroscopic (EELS) measurements were carried out at the edge of a Ru-NC NW, where four zones I–IV were selected (Fig. 2a): (I) ruthenium single atoms, (II) ruthenium nanoclusters, (III) ruthenium nanoparticles, and (IV) ruthenium-free carbon matrix. From the EELS spectra in Fig. 2b–e, one can see that both C (ca. 300 eV) and N (ca. 400 eV) were rather visible in all four zones, whereas Ru (ca. 460 eV, insets to Fig. 2b–e) appeared only in zones I–III but was absent in zone IV, and in comparison to the spectral background, the Ru signals became intensified from single atoms (zone I) to nanoclusters (zone II) and further to nanoparticles (zone III). This suggests that ruthenium was most likely stabilized by coordination to nitrogen and/or carbon in the matrix. Furthermore, the fact that no oxygen signal (ca. 523 eV) was detected indicates that the Ru-NC samples were largely unoxidized.

**Fig. 3:**
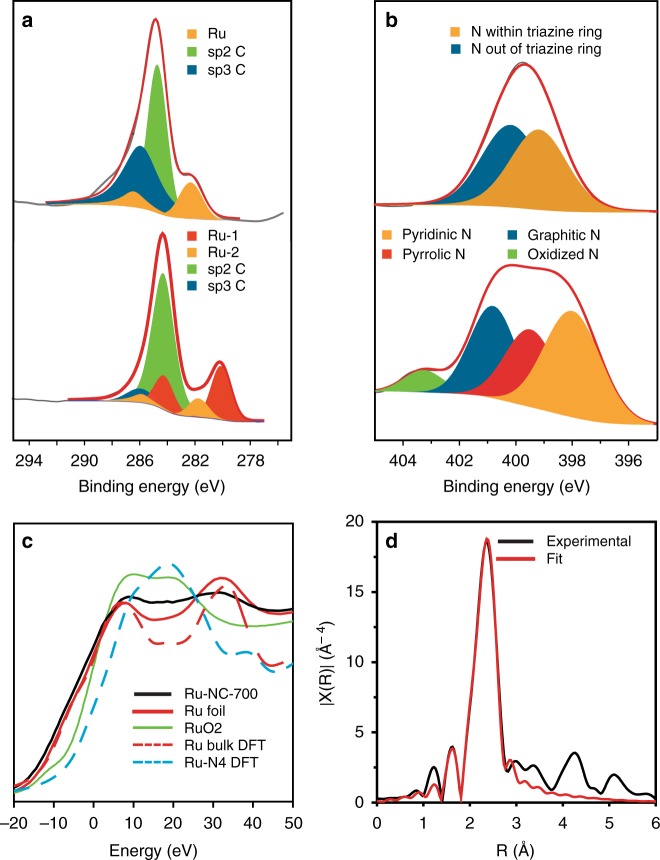
X-ray photoelectron spectroscopic and X-ray absorption spectroscopic studies. **a** C 1s and Ru 3d spectra of Ru-MF (upper panel) and Ru-NC-700 (lower panel). **b** N 1s spectra of Ru-MF (upper panel) and Ru-NC-700 (lower panel). Black curves are experimental data, shaded peaks are deconvolution fits, and red curves are the sum of the fits. **c** Normalized X-ray absorption near edge structure data for Ru-NC-700, solid lines are experimental data and dash lines are simulated data by density functional theory calculations. **d** Fourier transform of extended X-ray absorption fine structure data for Ru-NC-700, where black curve is experimental data and red curve is the best fit

Elemental analysis of the Ru-NC-*T* samples was then carried out by X-ray photoelectron spectroscopic (XPS) measurements (Fig. 3a, b and Supplementary Figure 5-8). The atomic ratio of Csp^2^/Csp^3^ increased markedly from ca. 1:1 for Ru-MF to 4.74:1 for Ru-NC-500, 4.22:1 for Ru-NC-600, 9.2:1 for Ru-NC-700, and 16.98:1 for Ru-NC-800, suggesting increasing graphitization with increasing pyrolysis temperature (Supplementary Table 1). In addition, two ruthenium species are resolved for the Ru-NC samples, the first one (red, Ru-1) can be ascribed to metallic Ru(0), whereas the other one (yellow, Ru-2) is at a somewhat higher energy, very close to Ru(II) in Ru-N coordination that was observed previously^27^. This is consistent with the formation of both ruthenium nanoparticles and ruthenium atomic species embedded within the carbon matrix, as manifested in TEM measurements (Figs. 1f and 2a and Supplementary Figure 4); and the atomic ratio of Ru-2/Ru-1 was found to decrease with increasing pyrolysis temperature, 0.45 for Ru-NC-500, 0.36 for Ru-NC-600, 0.35 for Ru-NC-700, and 0.31 for Ru-NC-800 (Supplementary Table 2). As the overall ruthenium content remained almost unchanged at ca. 4 at% among the Ru-NC-*T* series (Supplementary Table 3) and the nanoparticle core size increased from Ru-NC-500 to Ru-NC-800 (Fig. 1), this suggests that apparent nanoparticle sintering occurred with increasing pyrolysis temperature, whereas the number of atomic Ru species varied at a much lower rate. Nitrogen was also found to be doped into the carbon matrix of the Ru-NC samples (Fig. 3b and Supplementary Figure 8) in the forms of pyridinic, pyrrolic, graphitic, and oxidized N^40-42^, and with increasing pyrolysis temperature, the overall nitrogen content decreased accordingly, 5.24 at.% for Ru-NC-500, 3.44 at.% for Ru-NC-600, 2.04 at.% for Ru-NC-700, and 0.96 at.% for Ru-NC-800 (Supplementary Table 3). Additional data can be found in Supplementary Table 4-5, and more detailed discussion is included under Supplementary Figure 8.

Further structural insights were obtained from Raman and X-ray diffraction (XRD) measurements. Broad D and G bands can be observed at ca. 1350 and 1550 cm^−1^ in Raman measurements (Supplementary Figure 9), indicating defective structures of the carbon matrix. In addition, the vibrational bands at 400–600 cm^−1^ can be assigned to Ru-N/Ru-C stretching^43^, suggestive of the formation of RuC_*x*_N_*y*_ moieties in the samples. In XRD measurements (Supplementary Figure 10), all Ru-NC-*T* samples exhibited diffraction patterns that are consistent with those of *hcp* Ru. In addition, a broad peak can be identified within 2*θ* = 20°–30°, characteristic of the (002) diffraction of nanosized graphitic carbon^44^.

Consistent results were obtained from X-ray absorption spectroscopy (XAS) measurements. Figure 3c shows the X-ray absorption near-edge structure (XANES) spectra of Ru-NC-700, Ru foil, and RuO_2_. It can be seen that the absorption edge of Ru-NC-700 (black solid curve) is close to that of Ru foil (red solid curve) but markedly different from that of RuO_2_ (green solid curve), consistent with results from EELS measurements (Fig. 2) where no oxygen signals were detected. In addition, one can see that, within the range of 10–30 eV, the Ru-NC-700 sample shows an almost flat feature. This can be attributed to the combined contributions of Ru nanoparticles and single atoms, as Ru foil displays a valley (density functional theory (DFT) simulation of bulk Ru shows a consistent profile, red dashed curve), whereas Ru single atoms (e.g., RuN_4_, cyan dashed curve; more details in Supplementary Figure 11) contribute a peak from DFT XAS simulations. Figure 3d shows the Fourier transform X-ray absorption fine structure (FT-EXAFS) spectrum of Ru-NC-700, along with the theoretical fit. Two main peaks can be fitted by using a Ru-Ru and Ru-N/C (C and N are indistinguishable in the fitting) shell with a bond distance of 2.68 and 2.03 Å, respectively^45,46^. Moreover, from the fitting, the coordination number (CN) was estimated to be 6.6 for Ru-Ru and 1.3 for Ru-N/Ru-C (Supplementary Table 6). The former is markedly lower than that (12) of bulk Ru, likely due to the formation of ruthenium nanoparticles^47^, whereas the latter is abnormally large for the size of the nanoparticles observed in Fig. 1. These are consistent with the formation of both ruthenium nanoparticles and single atoms bonded to the N and/or C in the carbon matrix, as manifested in HAADF-STEM measurements in Figs. 1 and 2. Furthermore, based on the CN^47–50^, the Ru atomic ratio in single atoms vs nanoparticles was estimated to be ca. 0.30, very close to that (0.35, Supplementary Table 2) determined by XPS measurements (more detailed discussion in the Supporting Information under Supplementary Figure 11).

### Electrocatalytic activity toward HER

**Fig. 4:**
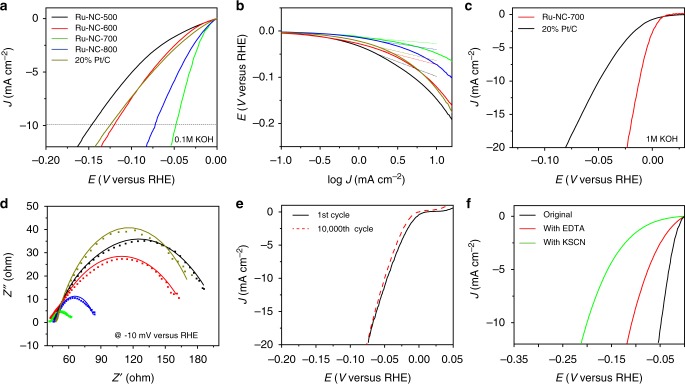
Electrochemical measurements. **a** Linear sweep voltammetric (LSV) curves of Ru-NC-*T* and commercial 20 wt% Pt/C in 0.1 M KOH. **b** Tafel plots of Ru-NC-*T* and 20% Pt/C. The legends are the same as those in **a**. **c** LSV curves of Ru-NC-700 and 20% Pt/C in 1 M KOH. **d** Nyquist plots of Ru-NC-*T* and 20% Pt/C in 0.1 M KOH at −10 mV vs. RHE. The legends are the same as those in **a**. Symbols are experimental data and solid curves are fits by Randles’ equivalent circuit. **e** Stability test of Ru-NC-700 in 0.1 M KOH, before and after 10,000 cycles within the potential window of −0.05 V to +0.05 V vs RHE. **f** Catalyst poisoning experiment for Ru-NC-700 in 0.1 M KOH with the addition of 10 mM of ethylenediaminetetraacetic acid (EDTA, red curve) or 10 mM of potassium thiocyanate (KSCN, green curve)

Remarkably, in electrochemical measurements, the Ru-NC-*T* samples exhibited significant HER activity in alkaline media, in comparison to commercial Pt/C. From Fig. 4a, it can be seen that in 0.1 M KOH, with increasingly negative electrode potentials, all Ru-NC-*T* samples exhibited nonzero cathodic currents, signifying apparent HER activity, and the activity varied among the samples. For instance, *η*_10_, an important parameter of the HER activity, can be identified at ‒146 mV for Ru-NC-500, ‒120 mV for Ru-NC-600, ‒47 mV for Ru-NC-700, and ‒72 mV for Ru-NC-800. Significantly, all but Ru-NC-500 showed an *η*_10_ that was even lower than that for commercial Pt/C (‒125 mV, Supplementary Table 7). Consistent behaviors can be seen in the Tafel plots in Fig. 4b, where the Tafel slope was only 24 mV dec^−1^ for Ru-NC-800, 14 mV dec^−1^ for Ru-NC-700, 43 mV dec^−1^ for Ru-NC-600, and 64 mV dec^−1^ for Ru-NC-500, in comparison to 39 mV dec^−1^ for Pt/C. This suggests that Ru-NC-700 and Ru-NC-800 samples even outperformed Pt/C toward HER in alkaline media, with Ru-NC-700 standing out as the best sample among the series. In 1 M KOH (Fig. 4c), the enhancement, as compared to Pt/C, is even more pronounced, with an *η*_10_ of only ‒12 mV for Ru-NC-700, much better than that of Pt/C (‒49 mV). In fact, to the best of our knowledge, the Ru-NC-700 sample outperforms any other Ru-based HER catalysts in basic media that have been reported in recent literature (Supplementary Table 8). In addition, Ru-NC-700 also showed a remarkably high HER activity in acid, with a low *η*_10_ of ‒29 mV and Tafel slope = 28 mV dec^−1^ in 0.5 M H_2_SO_4_, as compared to ‒13 mV and 18 mV dec^−1^ for Pt/C (Supplementary Figure 12).

The charge-transfer kinetics involved in HER were then examined by electrochemical impedance measurements. From the Nyquist plots acquired at −10 mV vs reversible hydrogen electrode (RHE) (Fig. 4d), one can see that all samples exhibited a quasi-semicircle, where the diameter represents the corresponding charge-transfer resistance (*R*_ct_). Interestingly, whereas *R*_ct_ of Ru-NC-500 (154 Ω) and Ru-NC-600 (129 Ω) was comparable to that of commercial Pt/C (136 Ω), it was significantly lower for Ru-NC-700 (20.7 Ω) and Ru-NC-800 (38.6 Ω) (Supplementary Table 7), in good agreement with results from voltammetric measurements (Fig. 4a). The Ru-NC-700 catalysts also exhibited remarkable stability. From Fig. 4e, it can be seen that there was virtually no change of the HER polarization curves after 10,000 potential cycles.

As both Ru nanoparticles and ruthenium single atoms (likely in Ru-N/Ru-C) are present in the Ru-NC-*T* samples (Fig. 1), EDTA and KSCN were used as poisoning species to help differentiate their contributions. From Fig. 4f, one can see that, with the addition of 10 mM KSCN, the *η*_10_ value shifted negatively by >150 mV, and the shift was much smaller (ca. 60 mV) when 10 mM EDTA was added to the solution. In fact, the HER performance of the EDTA-treated Ru-NC-700 sample was very similar to that with ruthenium nanoparticles deposited onto MF NWs (RuNP/MF, *η*_10_ = ‒124 mV, Supplementary Figure 13). Note that SCN^−^ readily adsorbs onto both Ru nanoparticle surface and Ru-N/Ru-C sites, and EDTA forms coordination bonds predominantly with the latter. The different poisoning effect observed above by SCN^−^ and EDTA (Fig. 4f) suggests that, whereas both Ru nanoparticles and Ru-N/Ru-C contributed to the HER activity, the latter likely played a dominant role. This is in sharp contrast with leading results in recent literatures where the HER activity was primarily attributed to ruthenium nanoparticles^18,30,31^.

This is further supported by results from another control experiment (Supplementary Figure 13), where the amount of RuCl_3_ added in sample synthesis was reduced to 1/8. The number of ruthenium nanoparticles in the resulting sample was diminished to about 1/30 of that for Ru-NC-700 (Supplementary Figure 13b-c), and XPS and XAS measurements showed a significant decrease of the Ru(0) fraction and an increase of the overall Ru oxidation states (Supplementary Figure 13d-g); yet, the HER performance (e.g., *η*_10_ = ‒50 mV) was almost identical to that of Ru-NC-700 (Supplementary Figure 13a). This implies only minimal contributions from ruthenium nanoparticles to the observed HER activity, and it is the ruthenium single atoms that dominated the HER performance (note that without the addition of RuCl_3_, the resulting N-doped carbon NWs, MF-700, essentially showed zero activity, Supplementary Figure 13a).

### Electrocatalytic active sites

**Fig. 5:**
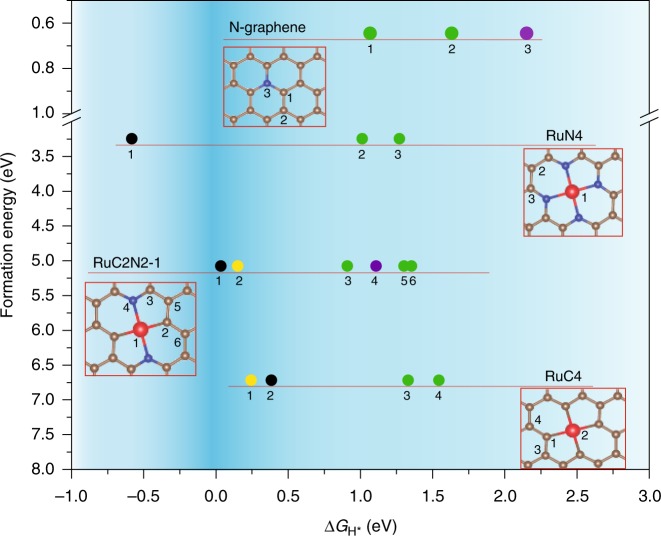
Energy diagram of different RuC_x_N_y_ structures from first principles calculations. The *x* axis is reaction free energy of hydrogen binding, where the negative values indicate strong binding while positive values indicate weak binding. The dark blue range indicates the candidate active sites with the best activity. The *y* Axis is formation energy of each configuration, where a more positive value signifies a structure that is more difficult to form. The colors denote candidate active sites: H binds to a ruthenium atom along the perpendicular direction to the plane (black), a carbon atom next to the ruthenium (yellow), a carbon atom next to nitrogen (green), or a carbon atom far away from ruthenium and nitrogen atoms (purple). The note records the data point from a certain structure, number records the possible active site. The version with complete RuC_*x*_N_*y*_ configurations can be found in Supplementary Figure 14 and the structures can be found in Supplementary Figure 15

Results from the above experimental studies showed that Ru-N/Ru-C moieties (i.e., single atom sites) played a dominant role in the HER performance, while Ru nanoparticles made only minor contributions. To identify the chemical configurations of the Ru-N/Ru-C moieties, we examined and compared the hydrogen binding energy with a range of RuC_*x*_N_*y*_ structures (*x* + *y* ≤ 4) involving 2, 3, or 4 coordinates (Supplementary Figure 14-15 and Supplementary Table 9)^28,32,51,52^. Among these, the data points of select stable structures for H binding are shown in Fig. 5, and unstable configurations are labeled in red color in Supplementary Table 9. From Fig. 5, one can see that the optimal configurations are those located in the upper portion of the dark blue region that corresponds to a low formation energy and close to zero |Δ*G*_H*_|. It is interesting to note that, whereas the RuN_4_ moiety exhibits the lowest formation energy among the possible structural configurations of RuC_*x*_N_4−*x*_ **(**Supplementary Figure 14-15 and Supplementary Table 9-11)^32,51,53^, ca. 3 eV lower than that of RuC_4_ (Fig. 5), the large |Δ*G*_H*_| of the Ru and N sites suggest that neither of these is likely the catalytic active site (Supplementary Figure 16). By contrast, RuC_2_N_2_-1 (Fig. 5) represents the optimal structure for HER, featuring a relatively low formation energy and close to zero |Δ*G*_H*_| (<0.2 eV) for both Ru and adjacent C, suggesting that both of these atomic sites might serve as the catalytic active sites (more discussion can be found under Supplementary Figure 14 and 17). For comparison, the Δ*G*_H*_ was much higher at ‒0.5 to ‒0.6 eV for all facets of Ru nanoparticles^54^. This is consistent with the results presented above that atomically dispersed ruthenium was the dominant contributor to the observed HER activity, whereas only minimal contributions from ruthenium nanoparticles.

**Fig. 6:**
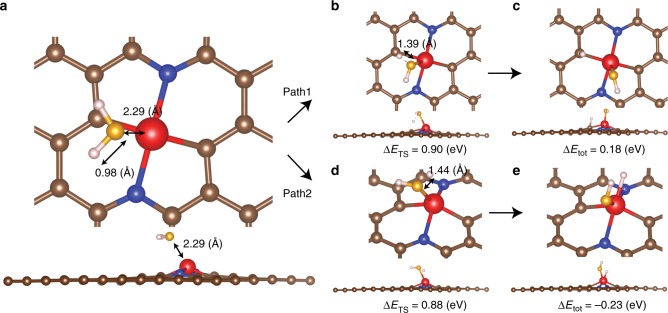
Reaction barriers and reaction pathways of Volmer’s step at RuC2N2-1. Note that the values in this figure are before solvation and entropy corrections to the free energies. Top view and side view of **a** initial state, **b**, **c** transition state and final state of reaction pathway 1, and **d**, **e** transition state and final state of reaction pathway 2. The brown, blue, red, yellow, and white sphere represent C, N, Ru, O, and H atoms, respectively

In addition, in previous studies^6,13,30^, it has been shown that the water dissociation process (Volmer reaction, step 1) is the rate-determining step for HER in alkaline media (Fig. 6). Therefore, understanding the reaction pathway and kinetic barriers is critical for uncovering the reaction mechanism. We computed the reaction barrier and transition state of the water dissociation step through the CI-NEB method^34^, as shown in Fig. 6 (more details can be found in the Supporting Information). Two possible pathways from the Volmer’s step are identified, with hydrogen binding to ruthenium or the carbons adjacent to ruthenium, respectively (the process can be better seen in Supplementary Movies 1 and 2). The corresponding reaction barriers along the configuration coordinates are shown in Supplementary Figure 18. In the initial state, after geometric optimization, the oxygen atom of H_2_O points to ruthenium at a distance of 2.29 Å (Fig. 6a). Then, in pathway 1, oxygen binds to the ruthenium atom, and one of the O-H bonds becomes elongated from 0.98 Å to 1.39 Å, as a transition state that has an energy 0.90 eV higher than the initial state (Fig. 6b). After entropy correction, zero-point energy correction, and solvation energy correction^55^, this pathway shows an energy barrier of 0.68 eV referenced to bulk water (details in Supplementary Table 12-13). Finally, the OH group settles onto the Ru atom, while H binds to an adjacent carbon atom (Fig. 6c). This structure shows an energy 0.18 eV higher than that of the initial state, indicating that pathway 1 is slightly unfavorable for H binding. In pathway 2, oxygen approaches the ruthenium site, and one of the hydrogen atoms also turns to bind. This transition state is shown in Fig. 6d. The total energy is 0.88 eV higher than that of the initial state; after corrections, the reaction barrier is only 0.59 eV, suggesting a favorable process for HER. Consistently, with OH and H binding to the same ruthenium site, the final state (Fig. 6e) is ‒0.23 eV lower than that of the initial state, indicative of favorable of H binding. Taken together, we can see that these two pathways exhibit similar reaction barriers and hence similar kinetic rates, and both the ruthenium and neighboring carbon most likely serve as the active sites, consistent with results from calculations discussed above. Remarkably, the reaction barrier of the structure is comparable to results in previous studies with Pt single atom catalysts (0.5–0.8 eV), but lower than that for Pt nanoparticles (0.8–1.0 eV)^30,56^, indicating competitive HER activity by such carbon-based catalysts, as manifested in experimental studies. We also computed the grand free energy under cathodic condition at a constant negative electrochemical potential along with the implicit solvation model^57^ (see the Computational method section for details). We found that neither the barriers nor the reaction energies varied appreciably (<∼0.1 eV), as compared to the results obtained under the constant charge condition (Supplementary Table 14), and the optimized geometry remained virtually identical.

## Discussion

In summary, Ru,N-codoped carbon NWs outperform commercial platinum toward HER in alkaline media. The remarkable performance is ascribed to the formation of RuC_*x*_N_*y*_ moieties, where the ruthenium centers as well as the C sites likely serve as the HER active centers, with hydrogen binding facilitated by the coordinating nitrogen sites. By contrast, contributions from ruthenium nanoparticles are minor. Results from this study can be exploited for the rational design and engineering of ruthenium-based single atom catalysts toward HER in alkaline media. In addition, this study highlights the significance of structural characterization at the atomic scale in unraveling the mechanistic origin of metal, nitrogen-codoped carbons in electrocatalytic reactions.

## Methods

### Chemicals

Sodium tellurite (Na_2_TeO_3_, 99.5%, Alfa Aesar), melamine (99% Acros Organics), hydrazine hydrate (N_2_H_4_, 64% v/v, Acros Organics), polyvinylpyrrolidone (PVP, K30, USB), sodium hydroxide (NaOH, Fisher Scientific), formaldehyde (37% v/v, Acros Organics), ammonia (NH_3_, 35% in water, Acros Organics), ruthenium(III) chloride (RuCl_3_, Strem Chemicals), and Pt/C (20 wt%, Alfa Aesar) were used as received without further purification. Water was supplied from a Barnstead Nanopure Water System (18.3 MΩ cm).

### Synthesis of Te NWs

Te NWs were prepared by a literature procedure^57^. Typically, 0.18 g of Na_2_TeO_3_ and 2 g of PVP were dissolved in 66 mL of Nanopure water under vigorous stirring to form a homogeneous solution, into which were then injected 6.7 mL of NH_3_ and 3.3 mL of N_2_H_4_. The solution was transferred to a 100 mL Teflon-lined autoclave container and heated at 180 °C for 3 h. The autoclave was then cooled down naturally, and the solution was stored in a 4 °C refrigerator.

### Synthesis of Te NWs–MF polymer core-sheath nanofibers

The preparation of nanofibers (Te-MF) has been described previously^42^. In brief, 10 mL of Te NWs was centrifuged at 3000 rpm for 2 min with the addition of acetone as a sinking agent. After washing by water and ethanol for 3 times, Te NWs were dispersed in 10 mL of water. Separately, 0.126 g of melamine in 10 mL of water was added into a 50 mL round-bottom flask, and the solution was heated at 90 °C under magnetic stirring, into which were then injected the Te NWs solution, 20 μL of 0.2 M NaOH, and 0.53 mL of formaldehyde. The solution was heated at 90 °C for 7 h before being cooled down naturally. The product was collected by centrifugation at 5000 rpm for 5 min, washed with water and ethanol, and dried in a vacuum chamber for 24 h.

### Synthesis of ruthenium and nitrogen codoped carbon NWs

In a typical experiment, 50 mg of Te-MF was dispersed into 30 mL of ethanol under magnetic stirring at 350 rpm and heated at 50 °C, into which was then added 40 mg of RuCl_3_. The color of the solution was found to change from blue to brown. The reaction was run overnight, and the solids were collected by centrifugation and vacuum-dried. The product (Ru-MF) was placed in a tube furnace and heated at a controlled temperature (500, 600, 700, and 800 °C) for 3 h at a heating rate of 5 °C min^−1^. The nitrogen flow was maintained at 200 cc min^−1^. The samples were denoted as Ru-NC-*T* with *T* = 500, 600, 700, or 800.

For the control experiment, 5 mg of RuCl_3_ (1/8 of the amount used for the synthesis of the above Ru-NC-*T* samples) was added instead while other conditions were kept the same, and the pyrolysis was carried out at 700 °C. This sample was referred to as Ru-NC-700 (1/8 Ru). In another control experiment, 5 mg of RuCl_3_ was reduced by an excess amount of NaBH_4_ in the presence of 10 mg of MF-700 under vigorous stirring overnight, where MF-700 was prepared by pyrolysis of Te-MF at 700 °C. The resulting sample was denoted as Ru NP/MF.

### Characterization

TEM measurements were carried out with a FEI Talos F200X high-resolution TEM. Double aberration-corrected HAADF-STEM measurements were carried out with a modified FEI Tiatan microscope (TEAM0.5) operated at 300 KV with a HAADF detector. The STEM probe semi-angle is 30 mrad, at a spatial resolution of 0.05 nm. EELS were acquired with a Nion U-HERMS200 microscope operated at 60 kV. Half convergence angle was set at 20 mrad, and a current was set at 150 pA with dispersion of 0.166 eV channel^−1^. The total integral time for spectral collection was 200 s. XRD studies were performed with a SmartLab 9 KW XRD system. Raman spectra were collected with a Thermo Electron Corporation DXR Microscope. XPS were acquired with a PHI-5702 XPS instrument.

### X-ray absorption spectroscopy

Powder samples were measured in X-ray fluorescence mode alongside a ruthenium foil reference. Ru K-edge EXAFS data were collected from the CLS@APS (Sector 20-BM) beamline at the Advanced Photon Source (operated at 7.0 GeV) in Argonne National Laboratory. All EXAFS measurements were conducted at room temperature under ambient pressure. EXAFS data were normalized and then transformed into *k*- and *R*-space using the Athena program and fitted with the Artemis program with conventional procedures^58^. A *k* weighting of 3, *k*-range of 2.5–13.0 Å^−1^, and a *R*-range of 1.4–2.8 Å was used for all the FT-EXAFS fitting analysis. In the fitting, both the *σ*^2^ and the *E*_0_ values of the two paths were correlated to minimize the number of independent variables to ensure a reliable fitting.

### Electrochemistry

Electrochemical measurements were carried out with a CHI 710 electrochemical workstation in a conventional three-electrode configuration. A Ag/AgCl electrode in 0.1 M KCl was used as the reference electrode and a graphite rod as the counter electrode. The reference electrode was calibrated against an RHE (Supplementary Figure 19), and all potentials in the present study were referred to this RHE. To prepare catalyst inks, 4 mg of the catalysts obtained above were added into 1 mL of ethanol and 10 μL of nafion solution under sonication to form a homogeneous dispersion. Ten μL of the ink was then dropcast onto a clean glassy carbon disk electrode (surface area 0.196 cm^2^) at a loading of 0.20 mg cm^−2^. Electrochemical impedance measurements were carried out with a Gamry Reference 600 instrument. IR-compensation was set at 85% of solution resistance in all measurements.

### Computational method

Computations were carried out with an open source planewave code Quantum ESPRESSO^59^. A 2D carbon matrix supercell was built on a 6 × 6 unit cell for a 4-coordinating Ru/N/C system and an 8 × 8 unit cell for other calculations. The vacuum was set as 14 Å to avoid periodic image interactions. The ultrasoft pseudopotential^60^ was adopted with kinetic and charge density cutoff at 40 and 240 Ry, respectively. A 2 × 2 × 1 Monkhorst–Pack *K*-point grid was sampled for the supercell. The total energy was converged to the accuracy of 1 meV per atom. The Marzari Vanderbilt smearing^61^ was adopted with the smearing parameter at 0.01 Ry. The electronic energy was converged to 10^−8^ Ry and the total force was converged to 10^−4^ a.u. Spin polarized calculations were considered for all systems. Density functional perturbation theory^62^ was employed to calculate the phonon frequency as inputs for entropy and zero-point energy. The implicit solvation energy correction was applied with a newly developed solvation model (CANDLE)^63^ that has been shown to be suitable for various surfaces, with the open source code JDFTx^64,65^. The energy barrier calculation was carried out by CI-NEB method^34^. The Van Der Waals correction was applied for the images with Grimme’s D2 scheme^66,67^. The calculation of XANES was performed using the XSpectra code^68^ in the Quantum Espresso package. The X-ray absorption cross-section is described by Fermi’s Golden rule in the single particle approximation with all-electron wavefunctions. The all-electron wavefunctions are reconstructed in the framework of projected augmented wave from solutions of isolated atoms. Two projectors of each quantum number l were included in PAW to properly reconstruct the all-electron wavefunctions. The final-state effect is included by generating a half-hole or a complete hole in the core of PAW. The spin–orbit coupling in final states (bands near Fermi levels) is neglected. The constant potential calculation is performed to obtain the grand free energy at the constant electrochemical potential along with the implicit solvation model, where the charged surfaces can be effectively screened by the ionic response in solution as implemented in JDFTx. Constant potential calculation was carried out at −0.01 V vs RHE at pH = 14^55,69^.

## Supplementary information

Supplementary Information

Description of Additional Supplementary Files

Supplementary Movie 1

Supplementary Movie 2

## Data Availability

All data presented in this study and the codes for first-principle calculations are available from the corresponding authors (S.C. and Y. Ping) upon request.
