# Supplementary material for: Ruthenium atomically dispersed in carbon outperforms platinum toward hydrogen evolution in alkaline media
Source: Nat Commun. 2019 Feb 7;10:631. doi: 10.1038/s41467-019-08419-3 (PMC6367462; doi:10.1038/s41467-019-08419-3)
Supplement: Supplementary file 1 — Supplementary Information [file 41467_2019_8419_MOESM1_ESM.pdf]

Supplementary information

# Ruthenium atomically dispersed in carbon outperforms platinum toward hydrogen evolution reaction in alkaline media

Lu et al.

**Supplementary Movie 1 Water dissociation dynamics.** Hydrogen binds to the carbon adjacent to ruthenium.

**Supplementary Movie 2 Water dissociation dynamics.** Hydrogen binds to ruthenium.

**Supplementary Table 1. C 1s binding energy and atomic ratio of $sp^2$ and $sp^3$ carbons by XPS measurements**

| Sample | $Csp^2$ | $Csp^3$ | $Csp^2/Csp^3$ atomic ratio |
|---|---|---|---|
| Ru-MF | 284.64 | 285.84 | 1 |
| Ru-NC-500 | 284.20 | 285.80 | 4.71 |
| Ru-NC-600 | 284.16 | 285.80 | 4.22 |
| Ru-NC-700 | 284.17 | 285.84 | 9.20 |
| Ru-NC-800 | 284.20 | 285.80 | 16.98 |

**Supplementary Table 2. Summary of Ru 3d binding energy (eV) by XPS measurements**

| Sample | Ru-1 ($3d_{5/2}$) | Ru-1 ($3d_{3/2}$) | Ru-2 ($3d_{5/2}$) | Ru-2 ($3d_{3/2}$) | Ru-2/Ru-1 |
|---|---|---|---|---|---|
| Ru-MF | 282.24 | 286.33 | 0 | 0 | |
| Ru-NC-500 | 280.18 | 284.28 | 281.87 | 285.97 | 0.45 |
| Ru-NC-600 | 280.06 | 284.16 | 281.75 | 285.75 | 0.36 |
| Ru-NC-700 | 280.04 | 284.14 | 281.66 | 285.76 | 0.35 |
| Ru-NC-800 | 280.00 | 284.10 | 281.50 | 285.60 | 0.31 |

**Supplementary Table 3. Elemental composition (at%) by XPS measurements**

| Sample | Ru | C | N | Te |
|---|---|---|---|---|
| **Ru-MF** | 1.48 | 88.56 | 9.61 | 0.34 |
| **Ru-NC-500** | 4.02 | 87.69 | 5.24 | 3.05 |
| **Ru-NC-600** | 4.66 | 89.38 | 3.44 | 2.51 |
| **Ru-NC-700** | 3.85 | 92.12 | 2.04 | 1.98 |
| **Ru-NC-800** | 3.89 | 93.03 | 0.96 | 2.11 |

**Supplementary Table 4. N 1s binding energy (BE, eV) and content by XPS measurements**

| Sample | Pyridinic | | Pyrrolic | | Graphitic | | Oxidized | |
|---|---|---|---|---|---|---|---|---|
| | BE (eV) | Content (%) | BE (eV) | Content (%) | BE (eV) | Content (%) | BE (eV) | Content (%) |
| Ru-MF | 399.16 (triazine ring) | | | | 400.16 (non-triazine ring) | | | |
| Ru-NC-500 | 398.27 | 53.1 | 399.62 | 19.0 | 400.66 | 11.8 | 402.11 | 16.1 |
| Ru-NC-600 | 398.06 | 49.3 | 399.47 | 23.4 | 400.84 | 17.1 | 402.50 | 10.1 |
| Ru-NC-700 | 398.00 | 39.5 | 399.47 | 26.2 | 400.80 | 28.5 | 403.27 | 5.8 |
| Ru-NC-800 | 398.00 | 25.9 | 399.50 | 29.8 | 400.80 | 37.3 | 402.50 | 6.9 |

**Supplementary Table 5. Te 3d binding energy (BE, eV) by XPS measurements**

| Sample | $3d_{5/2}$ | $3d_{3/2}$ |
|---|---|---|
| Ru-MF | 576.32 | 586.74 |
| Ru-NC-500 | 575.75 | 586.09 |
| Ru-NC-600 | 575.58 | 586.04 |
| Ru-NC-700 | 575.60 | 586.04 |
| Ru-NC-800 | 575.59 | 586.04 |

**Supplementary Table 6. EXAFS fitting results of Ru-NC-700 from Figure 3c and Ru-NC-700 (1/8) from Supplementary Figure 13g**

| | bond | Coordination number (CN) | Bond length R (Å) | $\sigma^2$ (Å) x$10^{-3}$ | $E_0$ (eV) | R factor |
|---|---|---|---|---|---|---|
| Ru-NC-700 | Ru-N/Ru-C | 1.3 (5) | 2.03 (2) | 4.2 (4) | -2.6 (9) | 0.0021 |
| | Ru-Ru | 6.6 (5) | 2.678 (3) | 4.2 (4) | -2.6 (9) | 0.0021 |
| Ru-NC-700 (1/8) | Ru-N/Ru-C | 2.4 (6) | 2.02 (1) | 8 (2) | -2 (1) | 0.0136 |
| | Ru-Ru | 6.4 (7) | 2.71 (1) | 8 (2) | -2 (1) | 0.0136 |
| Ru foil | Ru-Ru | 12 | 2.672(5) | 3.2(7) | -8(1) | 0.014 |

**Supplementary Table 7. Summary of HER performances in 0.1 M KOH**

| Sample | $R_{CT}$ (Ω) | Tafel slope (mV/dec) | $\eta_{10}$ (mV) |
|---|---|---|---|
| Ru-NC-500 | 154 | 64 | −146 |
| Ru-NC-600 | 129 | 43 | −120 |
| Ru-NC-700 | 20.7 | 14 | −47 |
| Ru-NC-800 | 38.6 | 24 | −72 |
| 20%Pt/C | 136 | 39 | −125 |

**Supplementary Table 8. Comparison of HER performance with results in recent literatures**

| catalyst | catalyst loading ($mg/cm^2$) | Electrolyte solution | $\eta_{10}$ (mV) | Reference |
|---|---|---|---|---|
| Ru-NC-700 nanowires | 0.2 | 0.1 M KOH | −47 | this work |
| | | 1 M KOH | −12 | |
| Ru@$C_2N$[1] | 0.285 | 1 M KOH | −17 | 1 |
| Pt-Ni nanowire[2] | 0.015 | 1 M KOH | −50~60 | 2 |
| Pt-Ni alloy[3] | ~0.66 | 0.1 M KOH | −65 | 3 |
| $Ni_2P$-Ru[4] | 0.28 | 1M KOH | −31 | 4 |
| Ru/$C_3N_4$/C[5] | 0.2 | 0.1M KOH | −79 | 5 |
| RuP2[6] | 1 | 1M KOH | −52 | 6 |
| IrCo@NC[7] | 0.285 | 1M KOH | −45 | 7 |
| RuCo@NC[8] | 0.275 | 1M KOH | −28 | 8 |
| Ru/NC[9] | 0.013 | 1M KOH | -21 | 9 |

**Supplementary Table 9. Hydrogen binding free energy at various candidate active sites. The legends are the same as those in Supplementary Figure 14. The red color indicates unstable structures, and the green color indicates possible active sites that have a preferred energy. 'Transferred to P-Ru' means that H is transferred to P-Ru spontaneously during geometrical optimization.**

| Ru Coordination number | Model | Candidate Active Site | Hydrogen binding free energy (eV) |
|---|---|---|---|
| 4 | RuN4 | P-Ru | -0.582943083 |
| 4 | RuN4 | 1-N | transferred to P-Ru |
| 4 | RuN4 | 2-C | 1.270275969 |
| 4 | RuN4 | 3-C | 1.013356014 |
| 4 | RuC1N3 | P-Ru | -0.703733128 |
| 4 | RuC1N3 | 1-N | transferred to P-Ru |
| 4 | RuC1N3 | 2-N | 0.97141363 |
| 4 | RuC1N3 | 3-N | 1.19205208 |
| 4 | RuC1N3 | 4-C | transferred to P-Ru |
| 4 | RuC1N3 | 5-C | 1.040701092 |
| 4 | RuC1N3 | 6-C | 1.083474449 |
| 4 | RuC1N3 | 7-C | 0.947660424 |
| 4 | RuC1N3 | 8-C | 1.124005844 |
| 4 | RuC1N3 | 9-C | 0.926125809 |
| 4 | RuC1N3 | 10-C | 0.959837379 |
| 4 | RuC2N2-1 | P-Ru | 0.033324738 |
| 4 | RuC2N2-1 | 1-N | 1.109348832 |
| 4 | RuC2N2-1 | 2-C | 0.910202129 |
| 4 | RuC2N2-1 | 3-C | 1.303215362 |
| 4 | RuC2N2-1 | 4-C (next to Ru) | 0.151619207 |
| 4 | RuC2N2-1 | 5-C | 1.35433012 |
| 4 | RuC2N2-2 | P-Ru | -0.562833968 |
| 4 | RuC2N2-2 | 1-N | 0.875459835 |
| 4 | RuC2N2-2 | 2-C | 1.0208236 |
| 4 | RuC2N2-2 | 3-C | 0.932096317 |
| 4 | RuC2N2-2 | 4-C | 0.987377088 |
| 4 | RuC2N2-2 | 5-C (next to Ru) | 0.082477116 |
| 4 | RuC2N2-3 | P-Ru | -0.609100653 |
| 4 | RuC2N2-3 | 1-N | 0.9531818 |
| 4 | RuC2N2-3 | 2-C | 1.120259527 |
| 4 | RuC2N2-3 | 3-C | 1.11597711 |
| 4 | RuC2N2-3 | 4-C | 0.930818312 |
| 4 | RuC2N2-3 | 5-C (next to Ru) | 0.248898807 |
| 4 | RuC2N2-3 | 6-C | 1.034132029 |
| 4 | RuC3N1 | P-Ru | -0.505320715 |
| 4 | RuC3N1 | 1-N | 0.606394933 |
| 4 | RuC3N1 | 2-C (next to Ru) | 0.249474165 |
| 4 | RuC3N1 | 3-C (next to Ru) | 0.096627094 |

| | | | |
|---|---|---|---|
| 4 | RuC3N1 | 4-C (next to Ru) | 0.210464709 |
| 4 | RuC3N1 | 5-C | 1.033313894 |
| 4 | RuC3N1 | 6-C | 1.389703657 |
| 4 | RuC3N1 | 7-C | 1.351560007 |
| 4 | RuC3N1 | 8-C | 0.839902045 |
| 4 | RuC3N1 | 9-C | 1.429662348 |
| 4 | RuC3N1 | 10-C | 1.229403111 |
| 4 | RuC4 | P-Ru | 0.38393673 |
| 4 | RuC4 | 1-C (next to Ru) | 0.243745845 |
| 4 | RuC4 | 2-C | 1.331248877 |
| 4 | RuC4 | 3-C | 1.54465848 |
| 3 | RuN3 | P-Ru | -0.679875991 |
| 3 | RuN3 | S-Ru | 0.174130811 |
| 3 | RuN3 | 1-N | transferred to P-Ru |
| 3 | RuN3 | 2-N | transferred to P-Ru |
| 3 | RuN3 | 3-N | transferred to P-Ru |
| 3 | RuN3 | 4-C | 1.633488252 |
| 3 | RuN3 | 5-C | 1.032694415 |
| 3 | RuN3 | 6-C | 1.331064723 |
| 3 | RuC1N2-1 | P-Ru | -0.66372827 |
| 3 | RuC1N2-1 | S-Ru | -0.075077231 |
| 3 | RuC1N2-1 | 1-C (next to Ru) | transferred to P-Ru |
| 3 | RuC1N2-1 | 2-N | 1.155161478 |
| 3 | RuC1N2-1 | 3-N | transferred to P-Ru |
| 3 | RuC1N2-1 | 4-C | 0.971755881 |
| 3 | RuC1N2-2 | P-Ru | -0.638631289 |
| 3 | RuC1N2-2 | S-Ru | transferred to P-Ru |
| 3 | RuC1N2-2 | 1-N | transferred to P-Ru |
| 3 | RuC1N2-2 | 2-C (next to Ru) | transferred to P-Ru |
| 3 | RuC1N2-2 | 3-N | transferred to P-Ru |
| 3 | RuC1N2-3 | P-Ru | -0.594901548 |
| 3 | RuC1N2-3 | S-Ru | transferred to P-Ru |
| 3 | RuC1N2-3 | 1-N | transferred to P-Ru |
| 3 | RuC1N2-3 | 2-N | transferred to P-Ru |
| 3 | RuC1N2-3 | 3-C (next to Ru) | transferred to P-Ru |
| 3 | RuC2N1-1 | P-Ru | -0.447719616 |
| 3 | RuC2N1-1 | S-Ru | transferred to P-Ru |
| 3 | RuC2N1-1 | 1-N | 0.639547772 |
| 3 | RuC2N1-1 | 2-C (next to Ru) | transferred to P-Ru |
| 3 | RuC2N1-1 | 3-C (next to Ru) | transferred to P-Ru |
| 3 | RuC2N1-2 | P-Ru | 0.190764095 |
| 3 | RuC2N1-2 | S-Ru | 0.167072966 |
| 3 | RuC2N1-2 | 1-C (next to Ru) | 0.157875539 |

| | | | |
|---|---|---|---|
| 3 | RuC2N1-2 | 2-N | 1.257627564 |
| 3 | RuC2N1-2 | 3-C (next to Ru) | 0.124481709 |
| 3 | RuC2N1-3 | P-Ru | -0.524735766 |
| 3 | RuC2N1-3 | S-Ru | transferred to P-Ru |
| 3 | RuC2N1-3 | 1-C (next to Ru) | -0.231514096 |
| 3 | RuC2N1-3 | 2-C (next to Ru) | transferred to P-Ru |
| 3 | RuC2N1-3 | 3-N | 0.590215326 |
| 3 | RuC3 | P-Ru | -0.1792363 |
| 3 | RuC3 | S-Ru | -0.089116372 |
| 3 | RuC3 | 1-C (next to Ru) | 0.049455235 |
| 3 | RuC3 | 2-C (next to Ru) | 0.04675622 |
| 3 | RuC3 | 3-C (next to Ru) | transferred to P-Ru |
| 2 | RuN2 | P-Ru | -0.722423256 |
| 2 | RuN2 | S-Ru | -0.300078452 |
| 2 | RuN2 | 1-N | transferred to P-Ru |
| 2 | RuN2 | 2-C | 1.549525339 |
| 2 | RuN2 | 3-C | 1.025811475 |
| 2 | RuC1N1 | P-Ru | -0.79196364 |
| 2 | RuC1N1 | S1-Ru | -0.18466765 |
| 2 | RuC1N1 | S2-Ru | transferred to P-Ru |
| 2 | RuC1N1 | 1-C (next to Ru) | transferred to P-Ru |
| 2 | RuC1N1 | 2-N | transferred to P-Ru |
| 2 | RuC2 | P-Ru | -0.779528822 |
| 2 | RuC2 | S-Ru | transferred to P-Ru |
| 2 | RuC2 | 1-C (next to Ru) | transferred to P-Ru |
| 2 | RuN2-2 | P-Ru | -0.519890686 |
| 2 | RuN2-2 | S-Ru | 0.253072105 |
| 2 | RuN2-2 | 1-N | transferred to P-Ru |
| 2 | RuN2-2 | 2-C | 1.36172083 |
| 2 | RuC1N1-2 | P-Ru | -0.648603315 |
| 2 | RuC1N1-2 | S1-Ru | 0.151241222 |
| 2 | RuC1N1-2 | S2-Ru | transferred to P-Ru |
| 2 | RuC1N1-2 | 1-C | transferred to P-Ru |
| 2 | RuC1N1-2 | 2-N | transferred to P-Ru |
| 2 | RuC2-2 | P-Ru | -0.649165629 |
| 2 | RuC2-2 | S-Ru | transferred to P-Ru |
| 2 | RuC2-2 | 1-C (next to Ru) | transferred to P-Ru |
| N/A | N-Graphene | 1-N | 2.154131339 |
| N/A | N-Graphene | 2-C | 1.070537906 |
| N/A | N-Graphene | 3-C | 1.635290556 |

**Supplementary Table 10. Total energy, formation energy and solvation energy for various configurations.**

| Configuration | Total energy (eV) | Formation energy (eV) | Solvation (eV) |
|---|---|---|---|
| RuN4 | -14053.88271 | 3.247309947 | 0.20206962 |
| RuC1N3 | -13934.9728 | 4.488247484 | 0.16350527 |
| RuC2N2-1 | -13816.71433 | 5.077748935 | 0.16350527 |
| RuC2N2-2 | -13816.21232 | 5.57975964 | 0.16350527 |
| RuC2N2-3 | -13816.44378 | 5.348291919 | 0.16350527 |
| RuC3N1 | -13698.07195 | 6.051154135 | 0.128220334 |
| RuC4 | -13579.73399 | 6.72014313 | 0.077945429 |
| RuN3 | -21830.68115 | 4.76550164 | 0.335250349 |
| RuC1N2-1 | -21711.82635 | 5.951330658 | 0.303315424 |
| RuC1N2-2 | -21712.55747 | 5.220210396 | 0.303315424 |
| RuC1N2-3 | -21711.9603 | 5.817383243 | 0.303315424 |
| RuC2N1-1 | -21593.88655 | 6.222157749 | 0.226661151 |
| RuC2N1-2 | -21593.87565 | 6.233056448 | 0.226661151 |
| RuC2N1-3 | -21593.57644 | 6.53226737 | 0.226661151 |
| RuC3 | -21475.47719 | 6.962544987 | 0.235213439 |
| RuN2 | -20821.07624 | 6.25112943 | 0.29231618 |
| RuC1N1 | -20703.06705 | 6.591348372 | 0.29231618 |
| RuC2 | -20584.72876 | 7.260671085 | 0.29231618 |
| RuN2-2 | -20680.27857 | 6.566522776 | 0.230167793 |
| RuC1N1-2 | -20562.16202 | 7.014099644 | 0.230167793 |
| RuC2-2 | -20443.96974 | 7.537415627 | 0.230167793 |
| Graphene-N | -11373.10418 | 0.648133417 | 0.183929655 |

**Supplementary Table 11. Total energy of configuration with H binding, phonon energy and solvation energy for the active site candidates that bind H stably.**

| Ru Coordination number | Model | Active Site Candidate | Sites-H Total energy (eV) | Phonon (eV) | Solvation (eV) |
|---|---|---|---|---|---|
| 4 | RuN4 | P-Ru | -14070.4849 | 0.194106971 | 0.174993926 |
| 4 | RuN4 | 2-C | -14068.7693 | 0.326344503 | 0.180415417 |
| 4 | RuN4 | 3-C | -14069.0263 | 0.326344503 | 0.180415417 |
| 4 | RuC1N3 | P-Ru | -13951.734 | 0.166932401 | 0.20181984 |
| 4 | RuC1N3 | 2-N | -13950.1782 | 0.352093487 | 0.136053296 |
| 4 | RuC1N3 | 3-N | -13949.9576 | 0.352093487 | 0.136053296 |
| 4 | RuC1N3 | 5-C | -13950.1283 | 0.326344503 | 0.181138457 |
| 4 | RuC1N3 | 6-C | -13950.0855 | 0.326344503 | 0.181138457 |
| 4 | RuC1N3 | 7-C | -13950.2213 | 0.326344503 | 0.181138457 |
| 4 | RuC1N3 | 8-C | -13950.045 | 0.326344503 | 0.181138457 |
| 4 | RuC1N3 | 9-C | -13950.2429 | 0.326344503 | 0.181138457 |

| | | | | | |
|---|---|---|---|---|---|
| 4 | RuC1N3 | 10-C | -13950.2092 | 0.326344503 | 0.181138457 |
| 4 | RuC2N2-1 | P-Ru | -13832.7385 | 0.166932401 | 0.20181984 |
| 4 | RuC2N2-1 | 1-N | -13831.7818 | 0.352093487 | 0.136053296 |
| 4 | RuC2N2-1 | 2-C | -13831.9921 | 0.326344503 | 0.172939105 |
| 4 | RuC2N2-1 | 3-C | -13831.5991 | 0.326344503 | 0.172939105 |
| 4 | RuC2N2-1 | 4-C (next to Ru) | -13832.74 | 0.307417048 | 0.181138457 |
| 4 | RuC2N2-1 | 5-C | -13831.548 | 0.326344503 | 0.172939105 |
| 4 | RuC2N2-2 | P-Ru | -13832.8326 | 0.166932401 | 0.20181984 |
| 4 | RuC2N2-2 | 1-N | -13831.5137 | 0.352093487 | 0.136053296 |
| 4 | RuC2N2-2 | 2-C | -13831.3795 | 0.326344503 | 0.172939105 |
| 4 | RuC2N2-2 | 3-C | -13831.4682 | 0.326344503 | 0.172939105 |
| 4 | RuC2N2-2 | 4-C | -13831.4129 | 0.326344503 | 0.172939105 |
| 4 | RuC2N2-2 | 5-C (next to Ru) | -13832.3071 | 0.307417048 | 0.181138457 |
| 4 | RuC2N2-3 | P-Ru | -13833.1103 | 0.166932401 | 0.20181984 |
| 4 | RuC2N2-3 | 1-N | -13831.6675 | 0.352093487 | 0.136053296 |
| 4 | RuC2N2-3 | 2-C | -13831.5115 | 0.326344503 | 0.172939105 |
| 4 | RuC2N2-3 | 3-C | -13831.5158 | 0.326344503 | 0.172939105 |
| 4 | RuC2N2-3 | 4-C | -13831.701 | 0.326344503 | 0.172939105 |
| 4 | RuC2N2-3 | 5-C (next to Ru) | -13832.3721 | 0.307417048 | 0.181138457 |
| 4 | RuC2N2-3 | 6-C | -13831.5976 | 0.326344503 | 0.172939105 |
| 4 | RuC3N1 | P-Ru | -13714.6291 | 0.166932401 | 0.160896703 |
| 4 | RuC3N1 | 1-N | -13713.6156 | 0.352093487 | 0.073998014 |
| 4 | RuC3N1 | 2-C (next to Ru) | -13714.027 | 0.307417048 | 0.173092876 |
| 4 | RuC3N1 | 3-C (next to Ru) | -13714.1798 | 0.307417048 | 0.173092876 |
| 4 | RuC3N1 | 4-C (next to Ru) | -13714.066 | 0.307417048 | 0.173092876 |
| 4 | RuC3N1 | 5-C | -13713.2621 | 0.326344503 | 0.173092876 |
| 4 | RuC3N1 | 6-C | -13712.9057 | 0.326344503 | 0.173092876 |
| 4 | RuC3N1 | 7-C | -13712.9438 | 0.326344503 | 0.173092876 |
| 4 | RuC3N1 | 8-C | -13713.4555 | 0.326344503 | 0.173092876 |
| 4 | RuC3N1 | 9-C | -13712.8657 | 0.326344503 | 0.173092876 |
| 4 | RuC3N1 | 10-C | -13713.066 | 0.326344503 | 0.173092876 |
| 4 | RuC4 | P-Ru | -13595.4882 | 0.15078184 | 0.213056832 |
| 4 | RuC4 | 1-C (next to Ru) | -13595.7051 | 0.314131392 | 0.126477587 |
| 4 | RuC4 | 2-C | -13594.6298 | 0.326344503 | 0.126477587 |
| 4 | RuC4 | 3-C | -13594.4164 | 0.326344503 | 0.126477587 |
| 3 | RuN3 | P-Ru | -21847.3803 | 0.205576313 | 0.29675007 |
| 3 | RuN3 | S-Ru | -21846.6078 | 0.207182634 | 0.37662551 |

| | | | | | |
|---|---|---|---|---|---|
| 3 | RuN3 | 4-C | -21845.2068 | 0.326344503 | 0.315834858 |
| 3 | RuN3 | 5-C | -21845.8076 | 0.326344503 | 0.315834858 |
| 3 | RuN3 | 6-C | -21845.5092 | 0.326344503 | 0.315834858 |
| 3 | RuC1N2-1 | P-Ru | -21728.5221 | 0.15078184 | 0.332371602 |
| 3 | RuC1N2-1 | S-Ru | -21727.9899 | 0.207182634 | 0.332371602 |
| 3 | RuC1N2-1 | 2-N | -21726.7905 | 0.352093487 | 0.218317583 |
| 3 | RuC1N2-1 | 4-C | -21726.9937 | 0.326344503 | 0.263878667 |
| 3 | RuC1N2-2 | P-Ru | -21729.2443 | 0.166932401 | 0.332371602 |
| 3 | RuC1N2-3 | P-Ru | -21728.6034 | 0.166932401 | 0.332371602 |
| 3 | RuC2N1-1 | P-Ru | -21610.483 | 0.166932401 | 0.356206262 |
| 3 | RuC2N1-1 | 1-N | -21609.3843 | 0.352093487 | 0.159613935 |
| 3 | RuC2N1-2 | P-Ru | -21609.8336 | 0.166932401 | 0.356206262 |
| 3 | RuC2N1-2 | S-Ru | -21609.8843 | 0.207182634 | 0.34301345 |
| 3 | RuC2N1-2 | 1-C (next to Ru) | -21609.9554 | 0.314131392 | 0.297967137 |
| 3 | RuC2N1-2 | 2-N | -21608.7553 | 0.352093487 | 0.159613935 |
| 3 | RuC2N1-2 | 3-C (next to Ru) | -21609.9888 | 0.314131392 | 0.297967137 |
| 3 | RuC2N1-3 | P-Ru | -21610.2499 | 0.166932401 | 0.356206262 |
| 3 | RuC2N1-3 | 1-C (next to Ru) | -21610.0456 | 0.314131392 | 0.297967137 |
| 3 | RuC2N1-3 | 3-N | -21609.1235 | 0.352093487 | 0.159613935 |
| 3 | RuC3 | P-Ru | -21491.722 | 0.15078184 | 0.297813969 |
| 3 | RuC3 | S-Ru | -21491.5601 | 0.219606873 | 0.157180457 |
| 3 | RuC3 | 1-C (next to Ru) | -21491.5544 | 0.303445414 | 0.206186346 |
| 3 | RuC3 | 2-C (next to Ru) | -21491.5571 | 0.303445414 | 0.206186346 |
| 2 | RuN2 | P-Ru | -20837.8146 | 0.205576313 | 0.250417096 |
| 2 | RuN2 | S-Ru | -20837.5044 | 0.207182634 | 0.361031572 |
| 2 | RuN2 | 3-C | -20835.648 | 0.326344503 | 0.235069265 |
| 2 | RuC1N1 | P-Ru | -20836.1717 | 0.326344503 | 0.235069265 |
| 2 | RuC1N1 | S1-Ru | -20719.8363 | 0.166932401 | 0.250417096 |
| 2 | RuC1N1 | S2-Ru | -20719.3798 | 0.207182634 | 0.361031572 |
| 2 | RuC2 | S-Ru | -20601.4694 | 0.15078184 | 0.250417096 |
| 2 | RuN2-2 | S-Ru | -20696.997 | 0.205576313 | 0.370923384 |
| 2 | RuN2-2 | 1-N | -20696.2257 | 0.207182634 | 0.370923384 |
| 2 | RuC1N1-2 | P-Ru | -20695.0382 | 0.326344503 | 0.235069265 |
| 2 | RuC1N1-2 | S1-Ru | -20578.9705 | 0.166932401 | 0.370923384 |
| 2 | RuC1N1-2 | S2-Ru | -20578.2109 | 0.207182634 | 0.370923384 |
| 2 | RuC2-2 | S-Ru | -20460.7627 | 0.15078184 | 0.370923384 |
| N/A | N-doped graphene | 1-N | -11386.9734 | 0.352093487 | 0.002929768 |

| | | | | | |
|---|---|---|---|---|---|
| N/A | N-doped graphene | 2-C | -11388.216 | 0.326344503 | 0.187725371 |
| N/A | N-doped graphene | 3-C | -11387.6512 | 0.326344503 | 0.187725371 |

**Supplementary Table 12. Correction of energy barrier of pathway-1**

| | **Total energy (eV)** | **Solvation (eV)** | **Phonon (eV, 298K)** | **A($H_2O$) (eV)** |
|---|---|---|---|---|
| Bare surface | -13820.76847 | 0.17839527 | 0 | |
| Transition state | -14292.21325 | 0.103716834 | 0.540467398 | |
| $H_2O$ | -471.6674915 | | | 0.012411 |
| | | | | |
| Barrier | 0.676089643 | | | |

**Supplementary Table 13. Correction of energy barrier of pathway-2**

| | **Total energy (eV)** | **Solvation (eV)** | **Phonon (eV, 298K)** | **A($H_2O$) eV** |
|---|---|---|---|---|
| Bare surface | -13820.76847 | 0.17839527 | 0 | |
| Transition state | -14292.22949 | 0.091087131 | 0.484105673 | |
| $H_2O$ | -471.6674915 | | | 0.012411 |
| | | | | |
| Barrier | 0.590852615 | | | |

A($H_2O$) is (free energy-total energy) of one water, including the entropy of rotation, translation, and vibration, and solvation energy of liquid water

**Supplementary Table 14. Result of constant potential calculation of barrier and reaction energy in Figure 6.** Note that the values in this table are before solvation and entropy corrections to the free energies.

| | **Constant potential at -10 mV** | | **Constant charge condition** | |
|---|---|---|---|---|
| | **Barrier eV** | **Reaction Energy eV** | **Barrier eV** | **Reaction energy eV** |
| Path1 | 0.94 | 0.25 | 0.9 | 0.18 |
| Path2 | 1.05 | -0.06 | 0.88 | -0.23 |

**Supplementary Note 1: Additional Details of Computational Studies**

The free energy of hydrogen binding is calculated by $\Delta G_{H^*} = \Delta E + \Delta TS - \Delta ZPE + \Delta E_{sol}$, where ΔE is the total energy difference, ΔTS is the entropy difference, ΔZPE is the zero-point energy difference and $\Delta E_{sol}$ is the solvation energy difference. The formation energy is calculated by $E_{formation} = E_{total} - \Sigma n_i \mu_i$, where n is the number of atom i, μ is the chemical potential of atom i. In this case, i can be carbon, nitrogen, ruthenium or hydrogen. The original raw data is shown in Supplementary Table 10 and Supplementary Table 11.

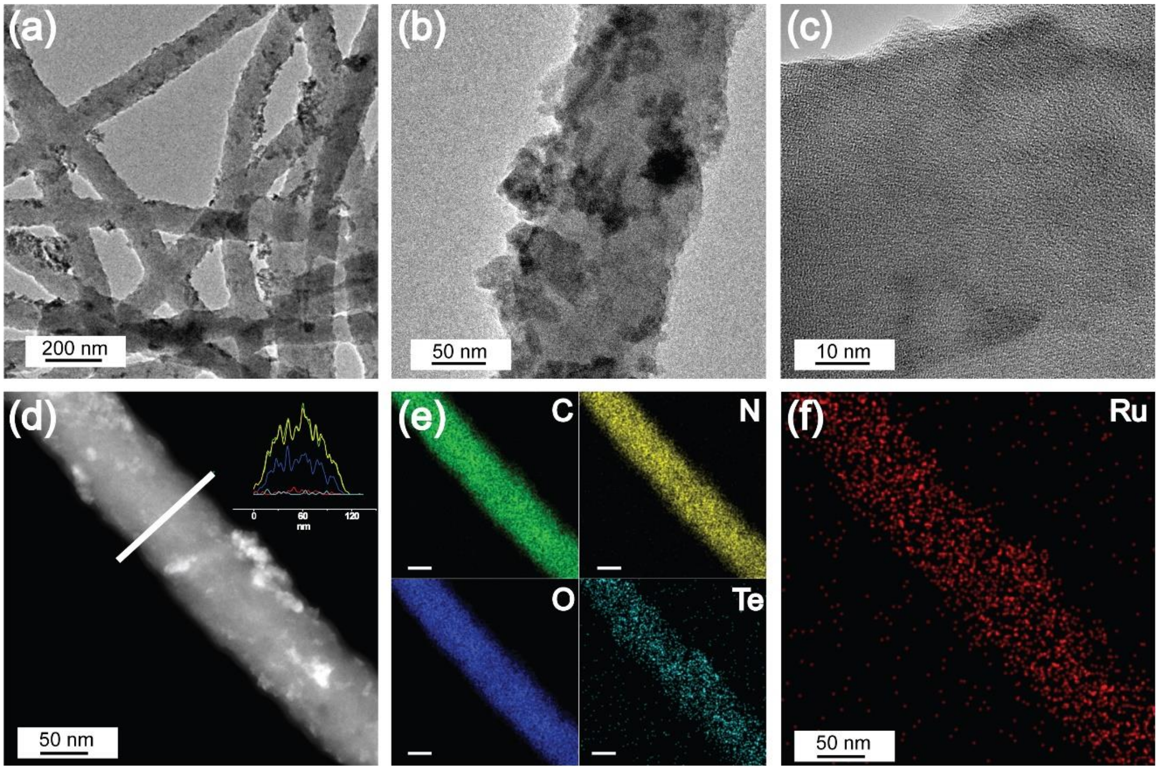


**Supplementary Figure 1 Transmission electron microscopy and elemental mapping studies.** (a)-(c) Representative TEM images and (d) HAADF-STEM image of Ru-MF. Inset is elemental line scans for C, N, O, Te and Ru, where the colors coincide with those in panels (e)-(f).

**Supplementary Note 2: Discussion of Energy-Dispersive X-ray Analysis**

It is difficult to use data from the EDX linear scans for the quantitative evaluation of elemental compositions. Therefore, the main conclusion from **Figure 1e-f** and **supplementary Figure 1d** is the identification of elements in the samples. A more reliable tool for quantitative elemental analysis is XPS, and the data are included in **Figure 3, supplementary Figure 5-8** and **supplementary Table 1-5**.

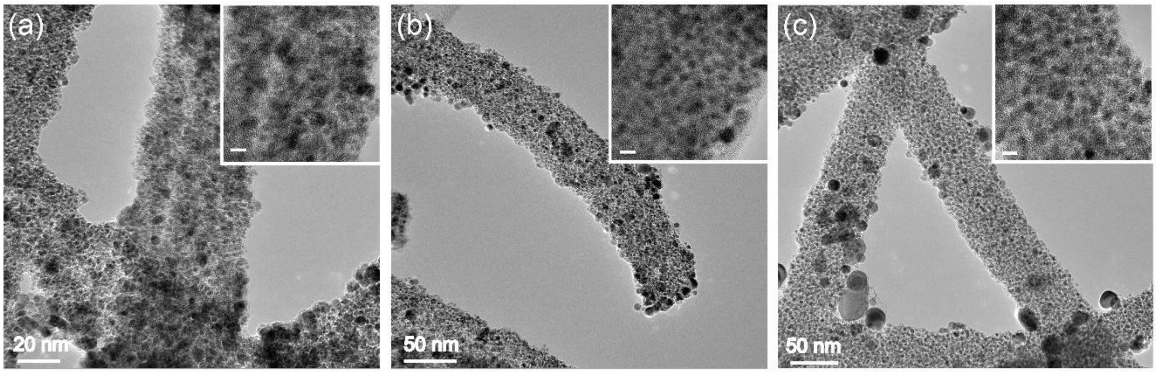


**Supplementary Figure 2 Transmission electron microscopy studies.** Representative TEM images of (a) Ru-NC-500, (b) Ru-NC-600, and (c) Ru-NC-700. Scale bars are (a) 20 nm, (b) 50 nm and (c) 50 nm. The insets are the corresponding images at higher magnification. Scale bars are all 5 nm

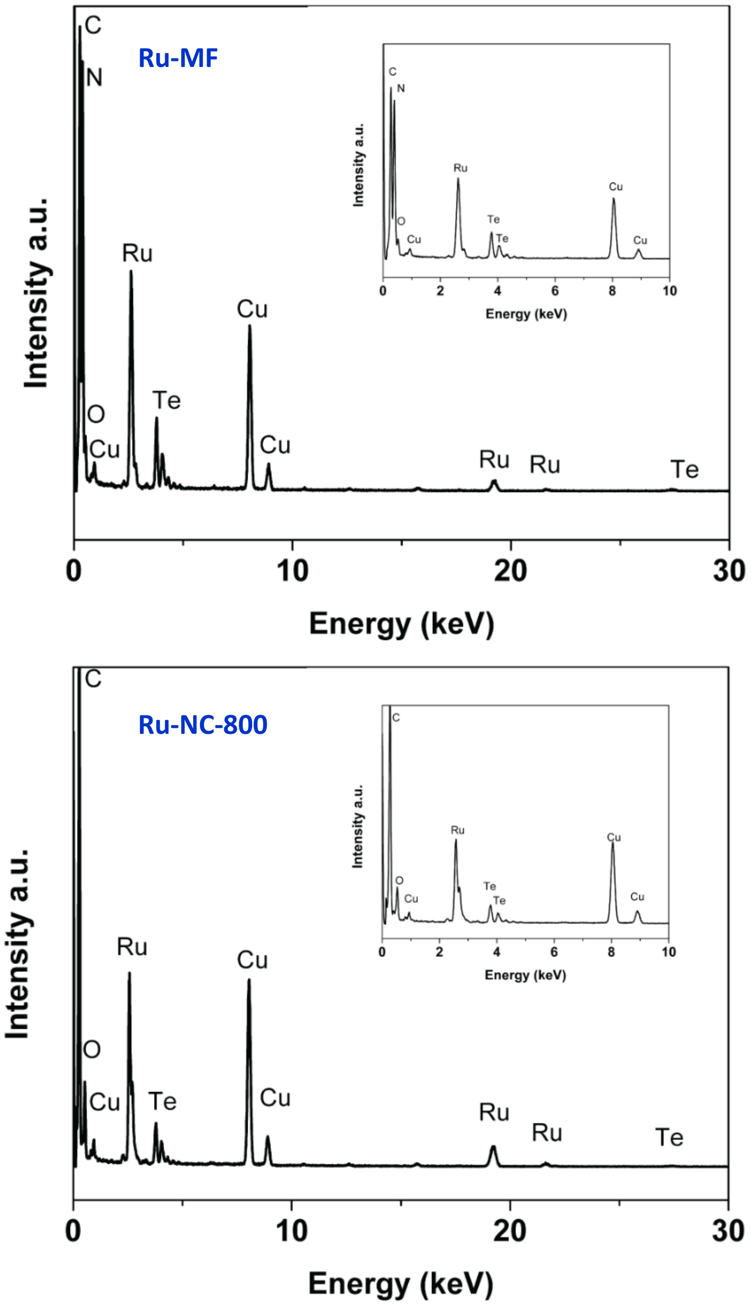


**Supplementary Figure 3 Energy-dispersive X-ray analysis**. EDX spectra of (top) Ru-MF and (bottom) Ru-NC-800. Insets is the zoom in of the regions between 0 and 10 eV. In both samples, the Cu peaks are from the TEM grids.

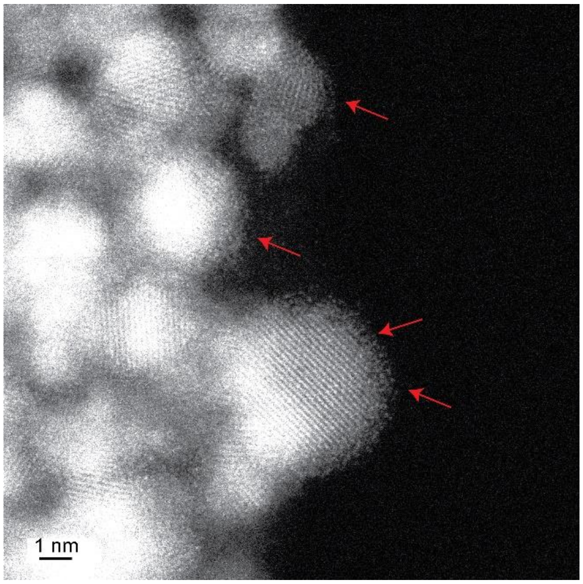


**Supplementary Figure 4 High-angle annular dark-field scanning transmission electron microscopy.** Representative 2AC-HAADF-STEM image of Ru-NC-700 of Ru nanoparticles and Ru single atoms. The arrows point out the carbon shell around Ru nanoparticles where Ru single atoms dispersed.

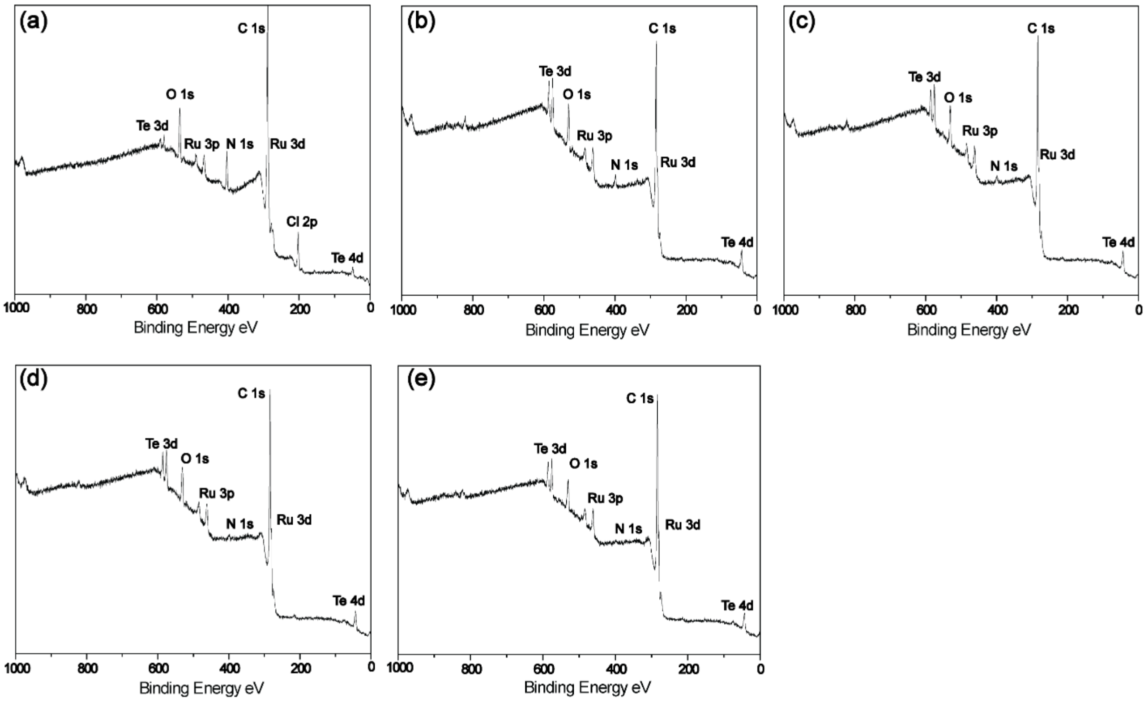


**Supplementary Figure 5 X-ray photoelectron spectroscopy studies**. XPS full survey spectra of (a) Ru-MF, (b) Ru-NC-500, (c) Ru-NC-600, (d) Ru-NC-700, and (e) Ru-NC-800.

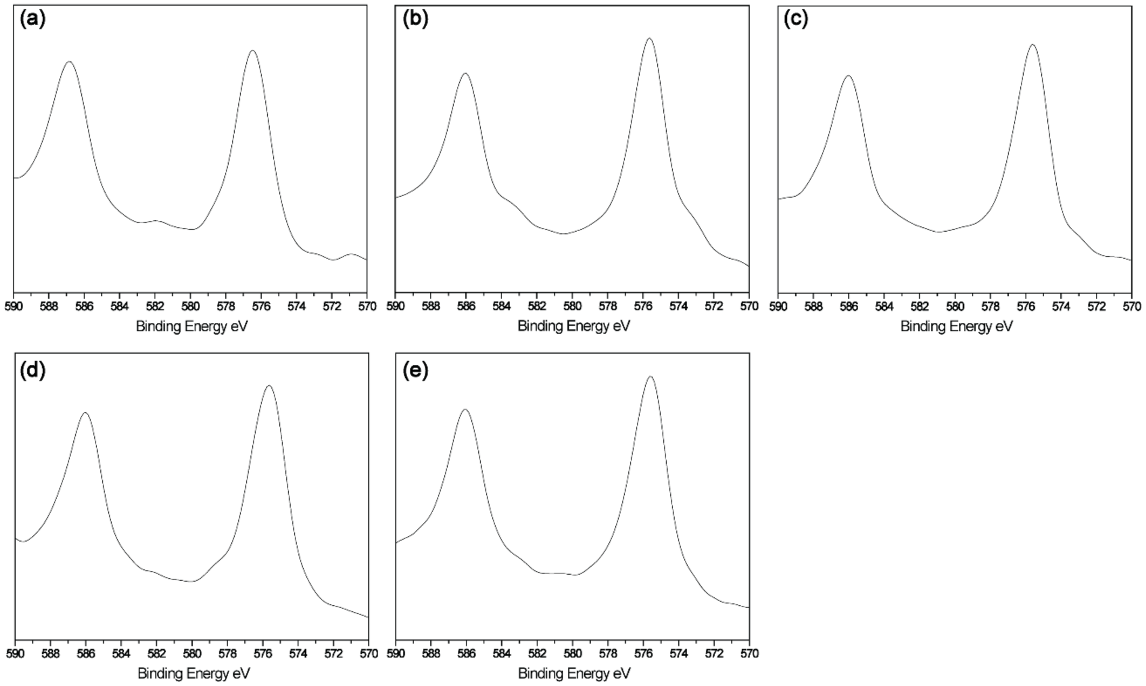


**Supplementary Figure 6 X-ray photoelectron spectroscopy studies.** XPS spectra of Te 3d electrons of (a) Ru-MF, (b) Ru-NC-500, (c) Ru-NC-600, (d) Ru-NC-700, and (e) Ru-NC-800.

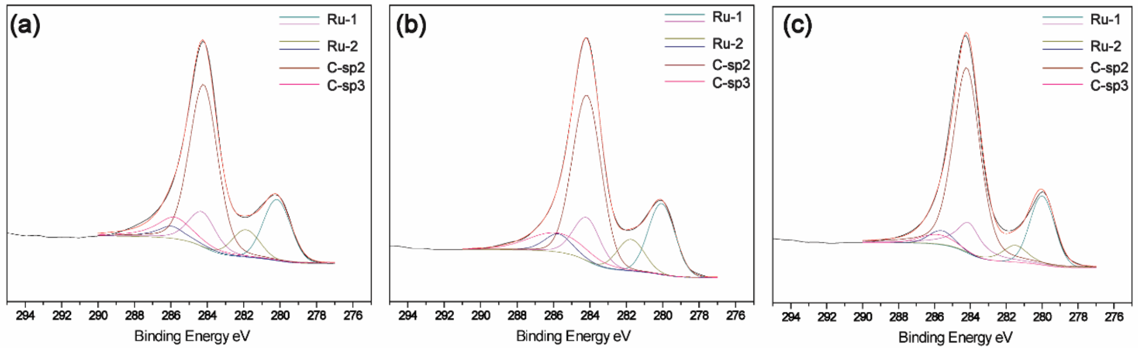


**Supplementary Figure 7 X-ray photoelectron spectroscopy studies.** XPS spectra of C 1s and Ru 3d electrons of (a) Ru-NC-500, (b) Ru-NC-600, and (c) Ru-NC-800. Black curves are experimental data and colored curves are deconvolution fits.

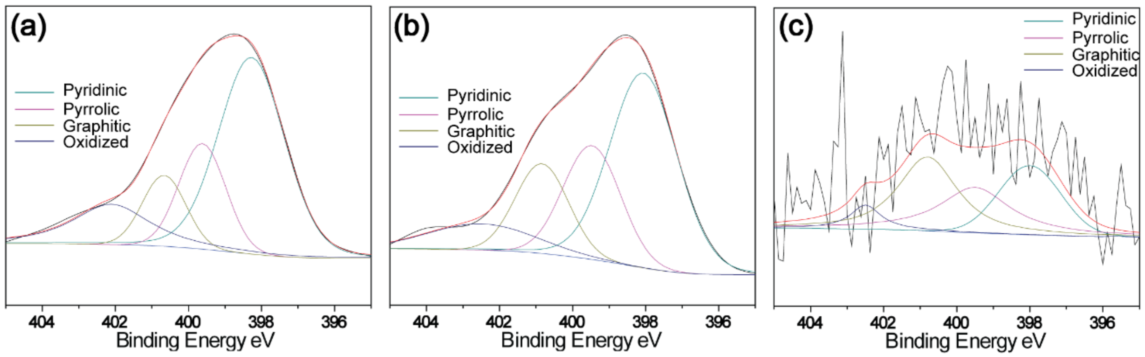


**Supplementary Figure 8 X-ray photoelectron spectroscopy studies.** XPS spectra of N 1s electrons of (a) Ru-NC-500, (b) Ru-NC-600, and (c) Ru-NC-800. Black curves are experimental data and colored curves are deconvolution fits.

**Supplementary Note 3: Additional Discussion of X-Ray Photoelectron Spectroscopy**

**Figure 3a** shows the XPS spectra of the C 1s and Ru 3d electrons of the Ru-MF and Ru-NC-700 samples. For the Ru-MF sample, two peaks can be resolved at 284.64 eV (green peak) and 285.84 eV (blue peak) with an atomic ratio of ca. 1:1, which are assigned to the $sp^2$ carbon within the triazine ring and $sp^3$ carbon out of the triazine ring, respectively. Similar $sp^2$and $sp^3$ carbons can be identified with the Ru-NC-700 sample, but the atomic ratio of $Csp^2/Csp^3$ increased markedly to 9.2:1. For comparison, this ratio was 4.74:1 for Ru-NC-500, 4.22:1 for Ru-NC-600, and 16.98:1 for Ru-NC-800,suggesting increasing graphitization with increasing pyrolysis temperature (**Supplementary Table 1**).The Ru-MF sample also shows a pair of peaks at 282.24 eV and 286.33 eV, which can be assigned to the 3d electrons of Ru(III) in $RuTe_x$ complexes (**Supplementary Figure 6**, and **Supplementary Table 2-3**). By contrast, for the Ru-NC-700 sample, two ruthenium species were resolved. The first pair (red, Ru-1) can be found at 280.04 and 284.14 eV and ascribed to Ru(0) in the nanoparticles, whereas the other pair (yellow, Ru-2) are at somewhat higher energies, 281.66 and 285.76 eV, very close to Ru(II) in Ru-N coordination that was observed previously[10]. This suggests that pyrolysis led to the formation of both ruthenium nanoparticles and ruthenium atomic species embedded within the carbon matrix, in good agreement with results from TEM measurements (**Figure 1f, 2a** and **Supplementary Figure 4**). Consistent results were obtained with other samples of the Ru-NC-T series (**Supplementary Figure 7**), where the atomic ratio of Ru-2/Ru-1 was found to decrease with increasing pyrolysis temperature, 0.45 for Ru-NC-500, 0.36 for Ru-NC-600, 0.35 for Ru-NC-700, and 0.31 for Ru-NC-800 (**Supplementary Table 2**). As the overall ruthenium content remained almost unchanged at ca. 4 at% among the Ru-NC-T series (**Supplementary Table 3**) and the nanoparticle core size increased from Ru-NC-500 to Ru-NC-800 (**Figure 1**), this suggests that apparent nanoparticle sintering occurred with increasing pyrolysis temperature, whereas the number of atomic Ru species varied at a much lower rate.

Nitrogen was also found to be doped into the carbon matrix. **Figure 3b** shows the XPS spectra of N 1s electrons of the Ru-MF and Ru-NC-700 samples; and data for other samples are shown in **Supplementary Figure 8**. Two peaks can be resolved at 399.16 eV and 400.16 eV at the atomic ratio of ca. 1:1 for Ru-MF in the upper panel that are due to the nitrogen in and out of the triazine ring, respectively.[11] For Ru-NC-700 in the lower panel, four nitrogen peaks were resolved, pyridinic N at 398.0 eV, pyrrolic N at 399.5 eV, graphitic N at 400.8 eV, and oxidized N at 403.3 eV (**Supplementary Table 4**)[12,13]. Additionally, one can see that with increasing pyrolysis temperature, the overall nitrogen content decreased accordingly, 5.24 at.% for Ru-NC-500, 3.44 at.% for Ru-NC-600, 2.04 at.% for Ru-NC-700, 0.96 at.% for Ru-NC-800 (**Supplementary Table 3**). Of these, pyridinic N represented the major species of the N dopants, but the fraction decreased appreciably from 53.1% to 25.9%, whereas graphitic N increased from 11.8% to 37.3%

and pyrrolic N from 19.0% to 29.8% (**Supplementary Table 4**), which can be ascribed to the discrepancy of the thermal stability of the different dopant configurations, as observed previously[11,14,15].

In addition, we do not feel that $C_3N_4$ moieties were formed in the samples in the present study. It is well-known that the triazine ring of $C_3N_4$ will decompose when the sample is pyrolyzed at temperatures over 600 °C, and the best samples in the present study were prepared at a much higher temperature (700-800 °C) — note that in our previous studies with Ru- $C_3N_4$ (ref. 27), the samples were prepared at very low temperature (~ 100 °C). This is confirmed by the above XPS study (**Figure 3, supplementary Figure 5-8** and **supplementary Table 1-5**). Specifically, sp2 C is the dominant species and sp3 C accounts for only a small fraction in the Ru-NC-T samples, inconsistent with that expected of $C_3N_4$. Furthermore, none of the four N dopant configurations can be assigned to N in $C_3N_4$ (ref. 27). In addition, in ref. 27, we carried out DFT calculations to examine the HER activity of Ru- $C_3N_4$, and found that the absolute value of hydrogen binding energy was around 0.5 eV, which was too high to explain the remarkable performance of Ru-NC-T in the present study.

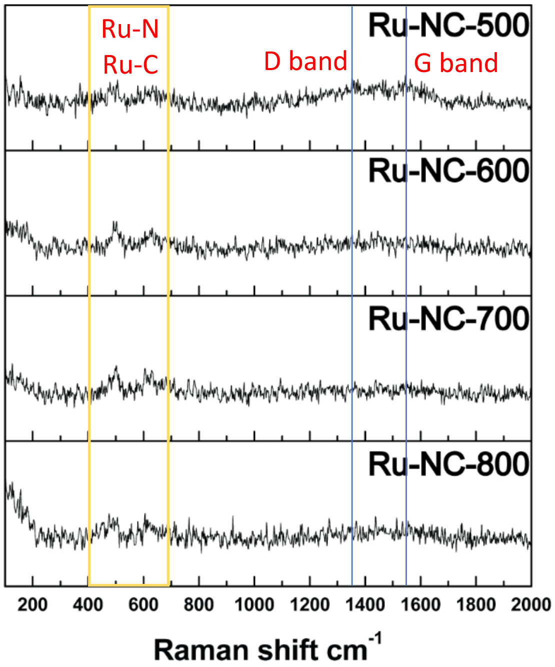


**Supplementary Figure 9 Raman spectroscopic studies**. Raman spectra of the Ru-NC-T samples

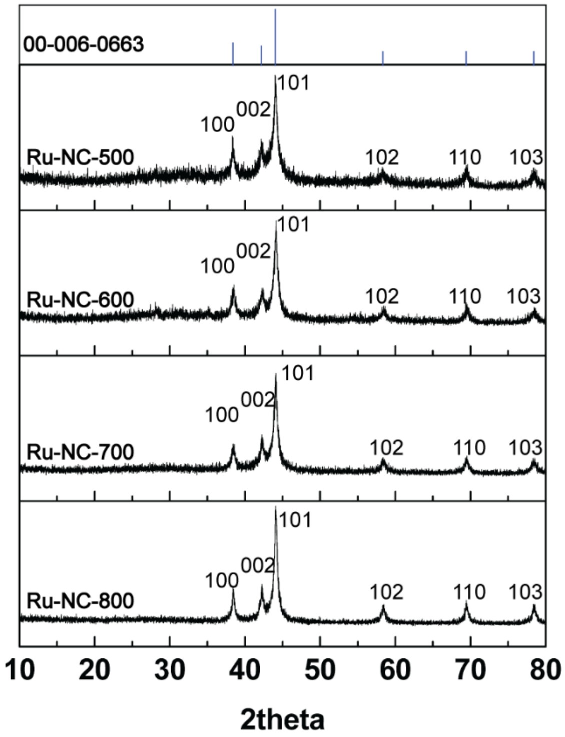


**Supplementary Figure 10 X-ray diffraction studies**. XRD profiles of Ru-NC-500, Ru-NC-600, Ru-NC-700 and Ru-NC-800. The standard profile of hcp Ru (00-006-0663) is included in the top of the figure.

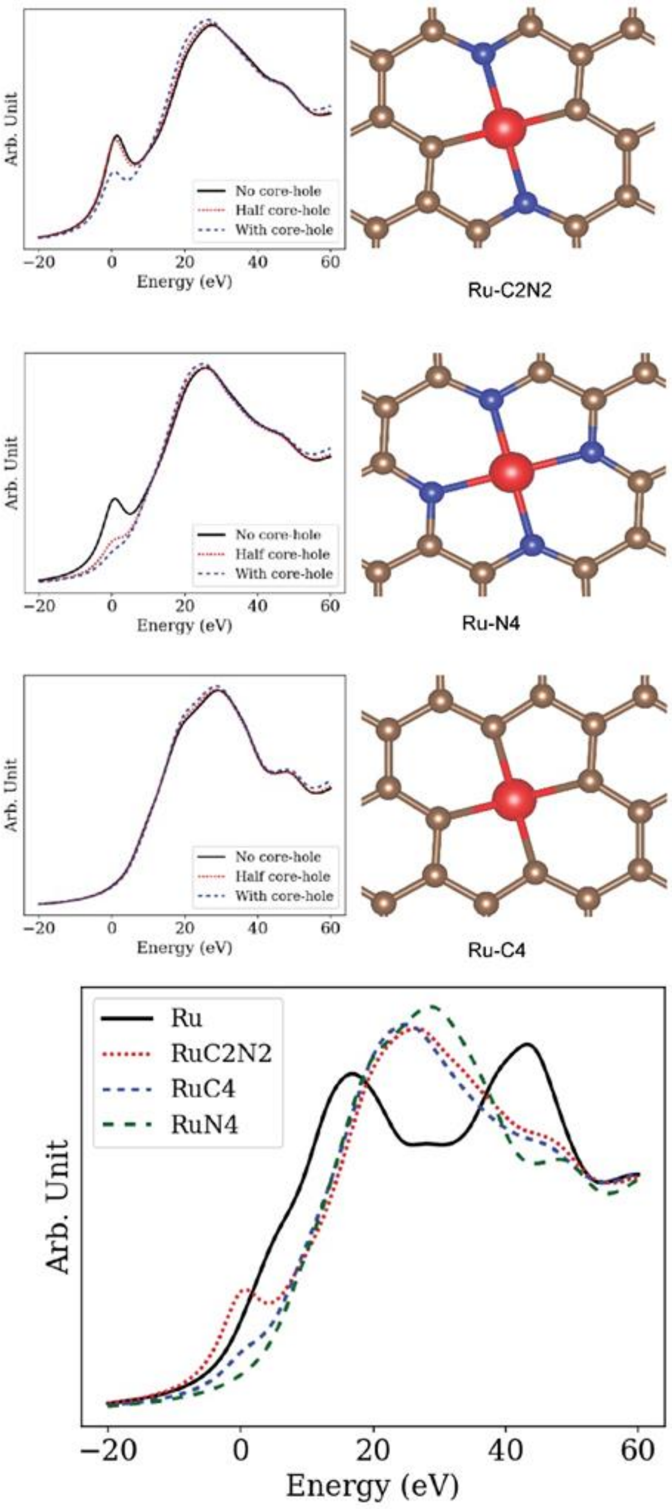


**Supplementary Figure 11a Simulated X-ray absorption near edge spectra**. Calculated XANES spectra of Ru K edge including core-hole effects for Ru NPs and Ru single atoms (e.g., $RuN_4$, $RuC_2N_2$ and $RuC_4$). To reduce the interactions between the absorbing Ru atoms, we use a supercell with 16 Ru atoms for Ru metal with k-point mesh 6 x 8 x 4, and a 6 x 6 graphene supercell with different defects for Ru-CN nanostructures with k-point mesh 2 x 2 x 1. The kinetic energy cutoff of wavefunction is set at 50 Ry and that of charge is 400 Ry. A smearing of 4 eV is chosen to reproduce the experimental spectra linewidth.

## Supplementary Note 4: Additional Discussion of X-Ray Adsorption Spectroscopy

**Additional discussion of EXAFS**: Fitting of the EXAFS data in **Figure 3d** yields a Ru-Ru CN of ca. 6.6 for Ru-NC-700, in comparison to 12 for bulk Ru, and a Ru-Ru bond distance (r) of 0.27 nm. From these, the average nanoparticle diameter (2R) can be estimated to be 0.87 nm by the Calvin equation (ref. 48), $CN_{nano} = \left[1 - \frac{3}{4}\left(\frac{r}{R}\right) + \frac{1}{16}\left(\frac{r}{R}\right)^3\right] CN_{bulk}$. The nanoparticle core diameter can also be estimated by the correlation between the first-shell coordination number and particle radius. From the calibration curve in **Supplementary Figure 11b** that was obtained by constructing a series of Ru *hcp* supercells of different sizes, a Ru-Ru coordination number of 6.6 corresponds to a nanoparticle diameter (2R) of ca. 1.2 nm.

It should be noted that these two values are lower than that obtained from TEM measurements (averaged 3.7 nm from **Figure 1**). This is likely because results obtained from EXAFS studies are a combined contribution of both Ru nanoparticles and nanoclusters (**Figure 2a**), whereas it is difficult to resolve small-sized nanoclusters in conventional TEM measurements (e.g., **Figure 1b-d**) where a large number of nanoparticles are counted to estimate the average core size.

Nevertheless, based on ref. 47, the first shell coordination number of a *hcp* Ru nanoparticle of 3.7 nm in diameter is estimated to be 8.5. That is, the fraction of ruthenium in nanoparticles is 6.6/8.5=0.77, corresponding to a Ru-2/Ru-1 atomic ratio of 0.30, very close to that obtained from XPS measurements (0.35, **Supplementary Table 2**).

**Additional X-ray absorption spectroscopy details.** A ruthenium foil is measured first to determine the $S_0^2$ value by fixing the coordination number to 12. The k-space and R-space of XAS are shown in Supplementary Figure 11c and d, where $S_0^2$ is estimated to be 0.78 in this study and adopted in further data analysis. The Ru-Ru and Ru-N coordination numbers are obtained by fixing the $S_0^2$ values, and the $E_0$ and $\sigma^2$ values are correlated together to reduce the number of independent variables in the fit. The coordination number and bond length values for both paths (Ru-Ru and Ru-N) are allowed to run free.

The data is analyzed by programs Artemis and Athena.

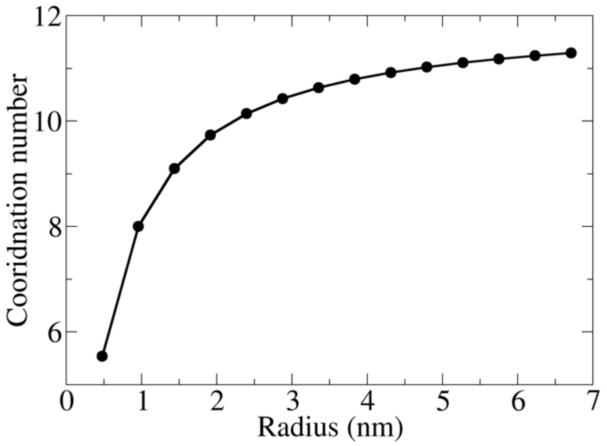


**Supplementary Figure 11b** Calibration plot of first-shell coordination number vs particle radius of *hcp* ruthenium nanoparticles.

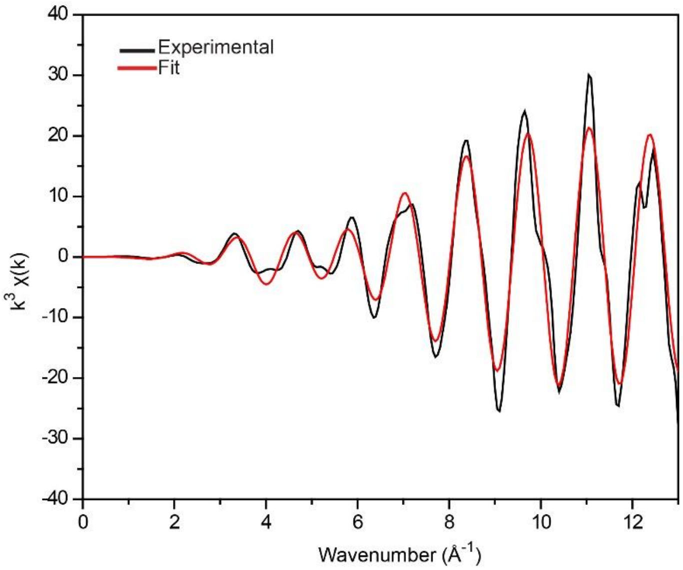


**Supplementary Figure 11c** k-space and best fit of XAS data of Ru foil**.**

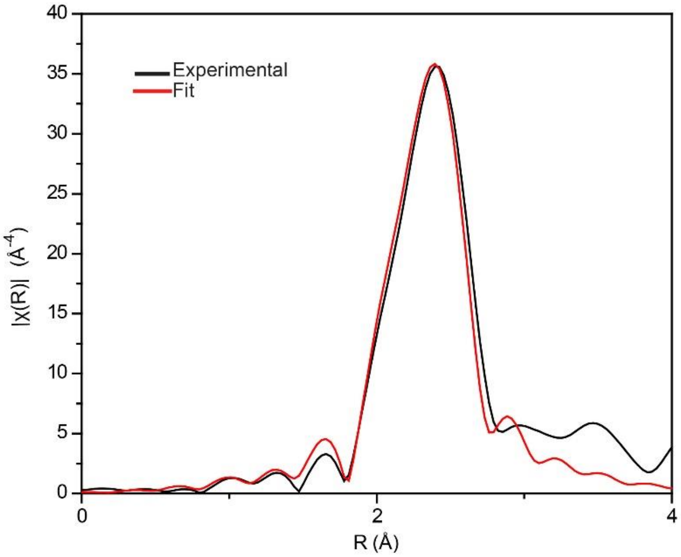


**Supplementary Figure 11d** R-space and best fit of XAS data of Ru foil.

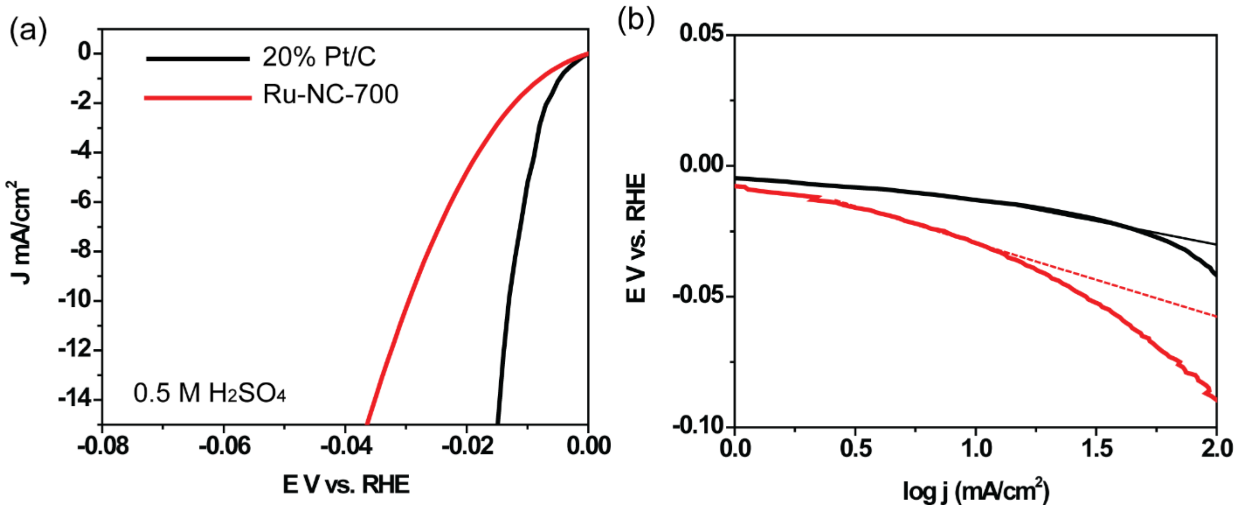


**Supplementary Figure 12 Electrochemical measurements in 0.5 M $H_2SO_4$**. (a) LSV curves of Ru-NC-700 and Pt/C. Potential scan rate 10 mV/s. (b) Tafel plot of Ru-NC-700 and Pt/C, the legends are the same as (a). Dashed curves are linear fits of the Tafel regions.

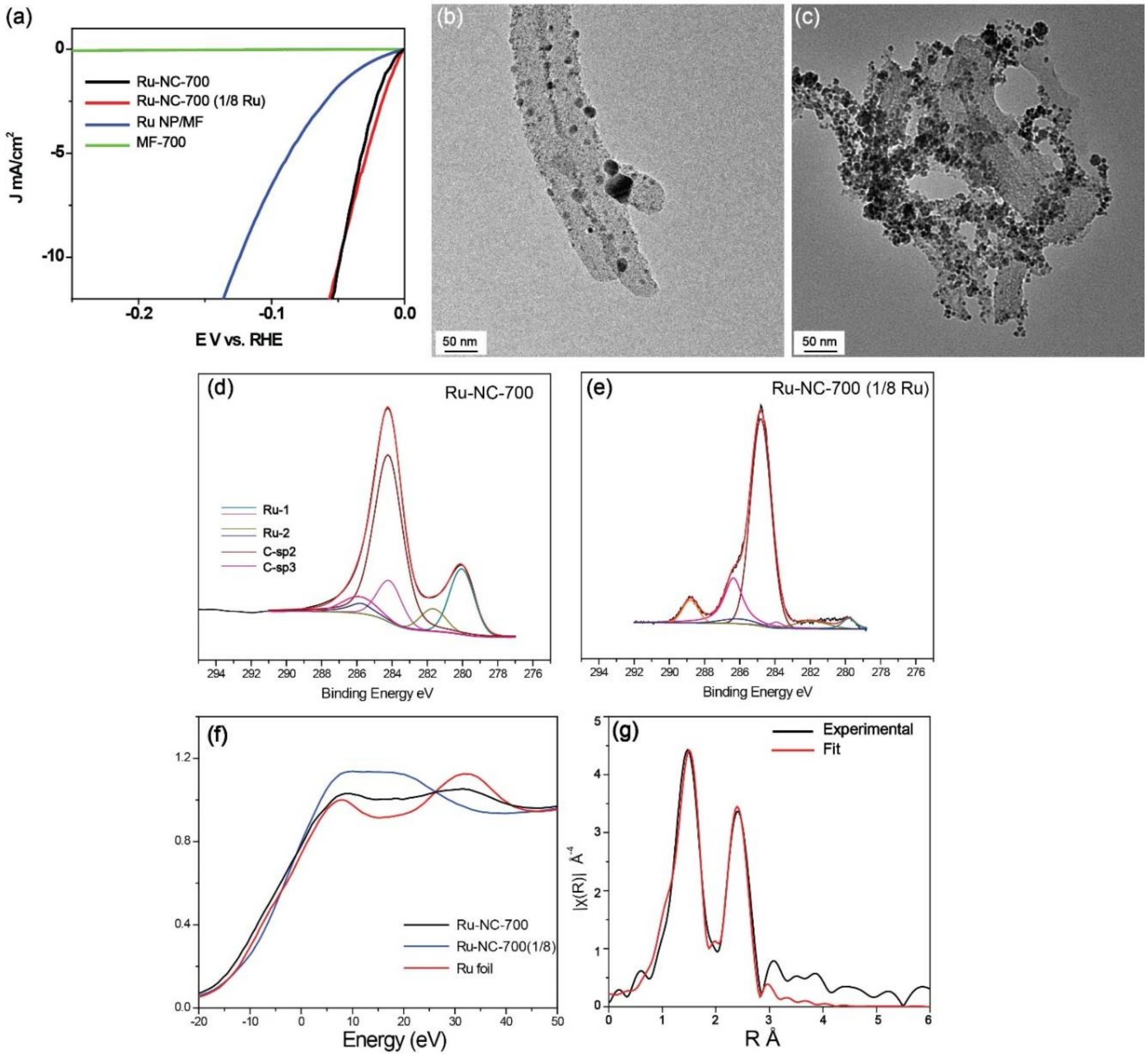


**Supplementary Figure 13 Control experiments.** (a) LSV curve of Ru-NC-700, Ru-NC-700 (1/8), Ru NP/MF, and MF-700 at the same loading in 0.1 M KOH. Potential scan rate 10 mV $s^{-1}$. Representative TEM images of (b) Ru-NC-700(1/8) and (c) Ru NP/MF. Scale bars are both 50 nm. (d) XPS of Ru-NC-700. (e) XPS of Ru-NC-700(1/8). (f) XANES data of Ru-NC-700, Ru-NC-700(1/8) and Ru foil. (g) EXAFS spectrum and its fit of Ru-NC-700(1/8).

**Supplementary Note 5: Additional Discussion of Control Experiments**

From the XPS data in **Supplementary Figure 13e**, it can be seen that the 1/8 Ru sample exhibited a much smaller Ru(0) peak, and a higher Ru-2/Ru-1 (single atom/nanoparticle) ratio of ca. 1.4:1, in comparison to only 0.35 for the Ru-NC-700 sample (**Supplementary Table 2**).

From the EXAFS data in **Supplementary Figure 13g,** it can be seen that in contrast to the Ru-NC-700 sample (**Figure 3d**), the Ru-Ru peak of the 1/8 sample is much weaker than the Ru-N/Ru/C peak. In addition, from the XANES data in **Supplementary Figure 13f**, one can see that a broad peak appears within the region of 10-30 eV, which is characteristic of atomic species. These observations are consistent with the formation of an increasing fraction of Ru atomic species and a higher overall (average) oxidation state of Ru.

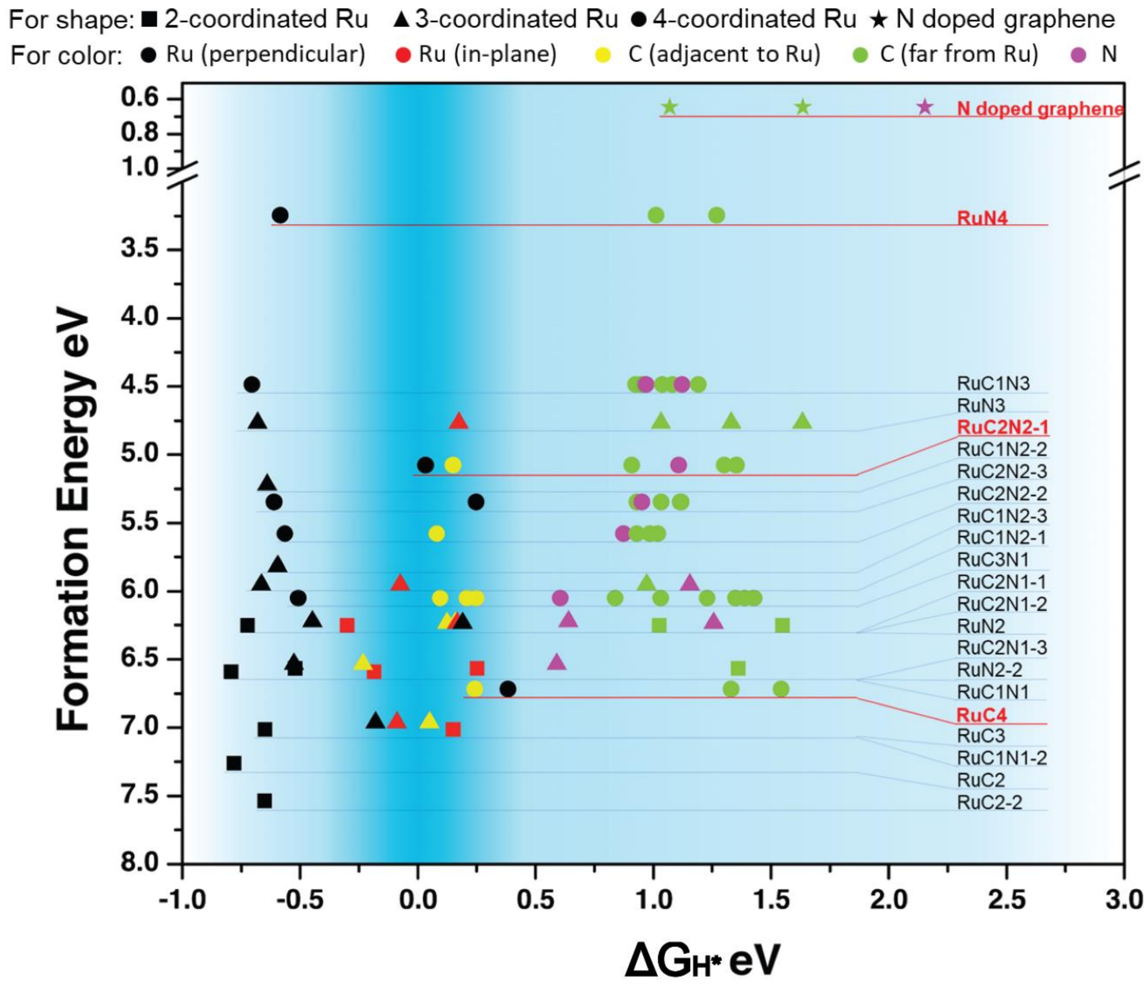


**Supplementary Figure 14 Energy diagram from first principles calculations of all possible $RuC_xN_y$ structures** depicted in **Supplementary Figure 15**. The X-axis is reaction free energy of hydrogen binding, the negative values indicate strong binding while positive values indicate weak binding. The dark blue range indicates the candidate active sites with the best activity. The Y-axis is formation energy of each configuration, where a more positive value signifies a structure that is more difficult to form. The square, triangle and circle symbols represent the 2-coordinate, 3-coordinate and 4-coordinate structures, while star represents the nitrogen doped carbon. The colors denote candidate active sites: H binds to a ruthenium atom along the perpendicular direction to the plane (black), in the plane (red), a carbon atom next to the ruthenium (yellow), a carbon atom next to nitrogen (green), or a carbon atom far away from ruthenium and nitrogen atoms (purple), respectively.

**Supplementary Note 6: Additional Discussion of Active Sites Analysis**

From **Supplementary Figure 14**, there are two points that warrant special attention here: (a) structural stability increases in the order of 2-coordinate< 3-coordinate< 4-coordinate; and (b) N-coordination leads to a lower formation energy than C-coordination. Note that whereas the $RuN_4$ moiety exhibits the lowest formation energy, ca. 3 eV lower than that of $RuC_4$, among the possible structural configurations of $RuC_xN_{4-x}$[11,16,17] the large $|\Delta G_{H^*}|$ of the Ru and N sites suggest that it is unlikely to be the catalytic active site (**Supplementary Figure 16**).

However, one can see from **Supplementary Figure 14** that none of the $RuN_4$ sites is located in the dark blue region, where hydrogen adsorption free energy ($\Delta G_{H*}$) is close to 0 ($|\Delta G_{H*}|$<0.5 eV), a condition favored for HER. For $RuN_4$, the Ru site shows a $\Delta G_{H*}$ of –0.58 eV, suggesting strong binding of H such that hydrogen intermediates are difficult to remove from the catalyst surface, whereas H binding to the nitrogen sites of $RuN_4$ is not stable, and the neighboring carbons to the $RuN_4$ sites exhibit a very high $\Delta G_{H*}$ of +1.0 to +1.3 eV, also energetically unfavorable for H binding. This result is consistent with Choi et al.'s work, which suggested that neither RuN4 nor RuC4 could be the active site.[17]

More favorable coordination configurations can be identified for HER from **Supplementary Figure 14**. First of all, when H binds to Ru in the 4-coordinated system of $RuC_{4-x}N_x$, H can only bind to Ru along the perpendicular direction (black) and the binding energy is too negative for most of the configurations. However, for the undersaturated $RuC_{3-x}N_x$and $RuC_{2-x}N_x$ systems, the low-coordinated Ru affords space for H binding to Ru in an in-plane fashion (red), where the $\Delta G_{H*}$ values are favorable for HER (the perpendicular and in-plan binding of H to Ru can be seen in **Supplementary Figure 17**). Second, the N sites can not only reduce the formation energy, but also stabilize in-plane H binding in the 2- and 3-coordinated systems, in particular, when H binds to the vacancy site opposite to N (e.g., S1 in RuC1N1-1 and RuC1N1-2 verses S2 in RuC1N1-1 and RuC1N1-2) (**Supplementary Figure 15** and **Supplementary Table 9**). Interestingly, it is unlikely that N is the active site, because it either binds H too weakly or cannot have stable binding (purple). Third, it can be seen that some carbons adjacent to Ru may have good activity (yellow). However, such active sites are stable only when the coordination number is 3 or 4, e.g., RuC2N2-1, RuC2N2-2, and RuC1N2-1, but not for the 2-coordinate systems. Other C sites show only weak H binding and are unlikely to be the HER active sites (green). Lastly, for Ru-free nitrogen-doped graphene (**Supplementary Figure 14**), it can be seen that both the N and C sites bind H too weakly, corresponding to low HER activity, in good agreement with experimental results (**Supplementary Figure 13**). In summary, for Ru,N-codoped carbons, the HER active sites are most likely the Ru atoms and/or adjacent C atoms.

From the candidate active sites (green in **Supplementary Table 9**), one can see that some even exhibit a binding energy close to zero, indicating that such configurations might contribute significantly to the high HER performance observed experimentally (**Figure 4**). Among these, we can see that RuC2N2-1 (**Supplementary Figure 14**) represents the optimal structure for high HER activity, considering a combination of low formation energy and close to zero $\Delta G_{H*}$. Notably, in a recent study[18], $\Delta G_{H*}$ was estimated to be –0.5 to –0.6 eV for all facets of Ru nanoparticles, which is much less favorable for HER than the active sites of $RuC_xN_y$ structures discussed above where $|\Delta G_{H*}|$ < 0.2 eV (**Supplementary Figure 14).**

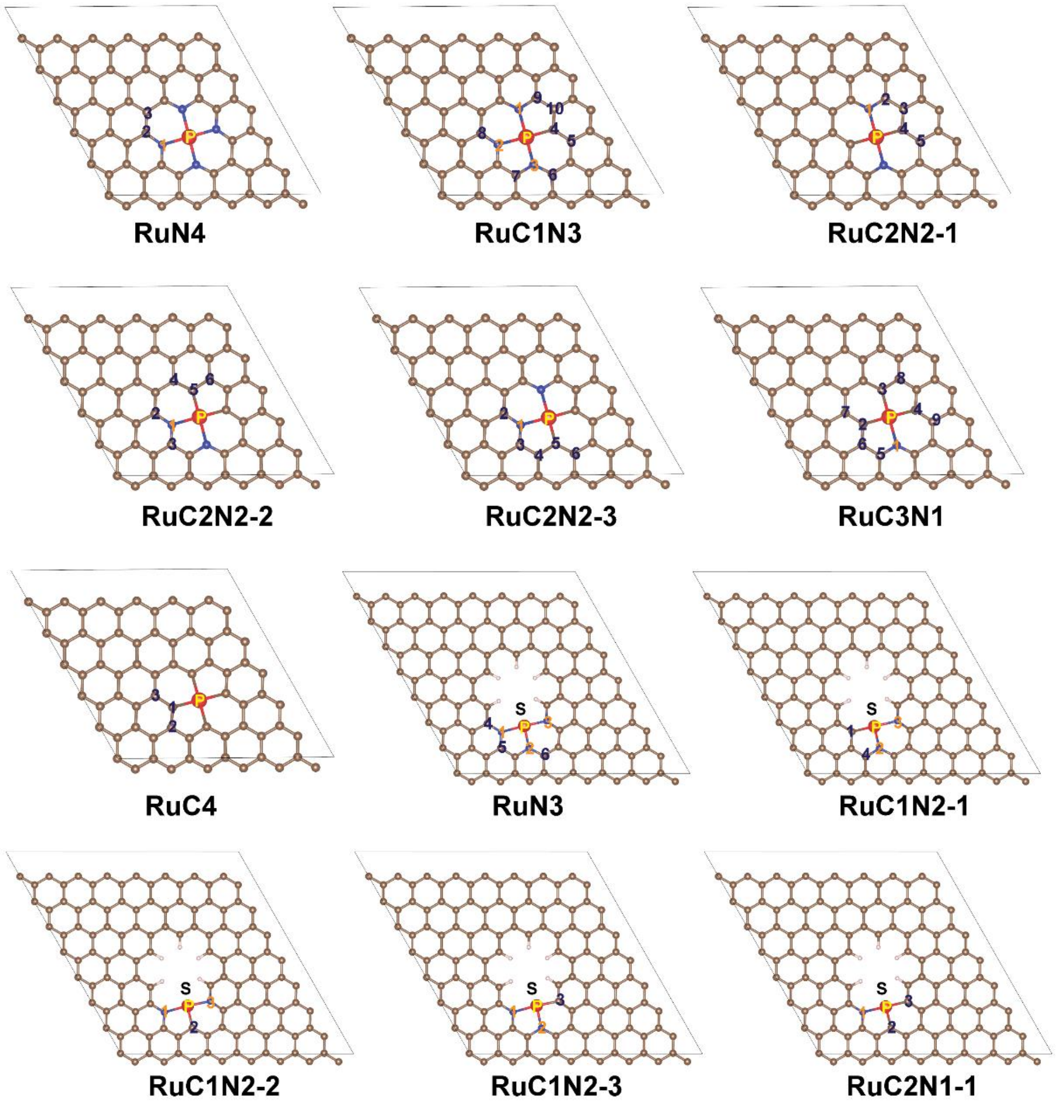


(supplementary Figure 15, continued on next page)

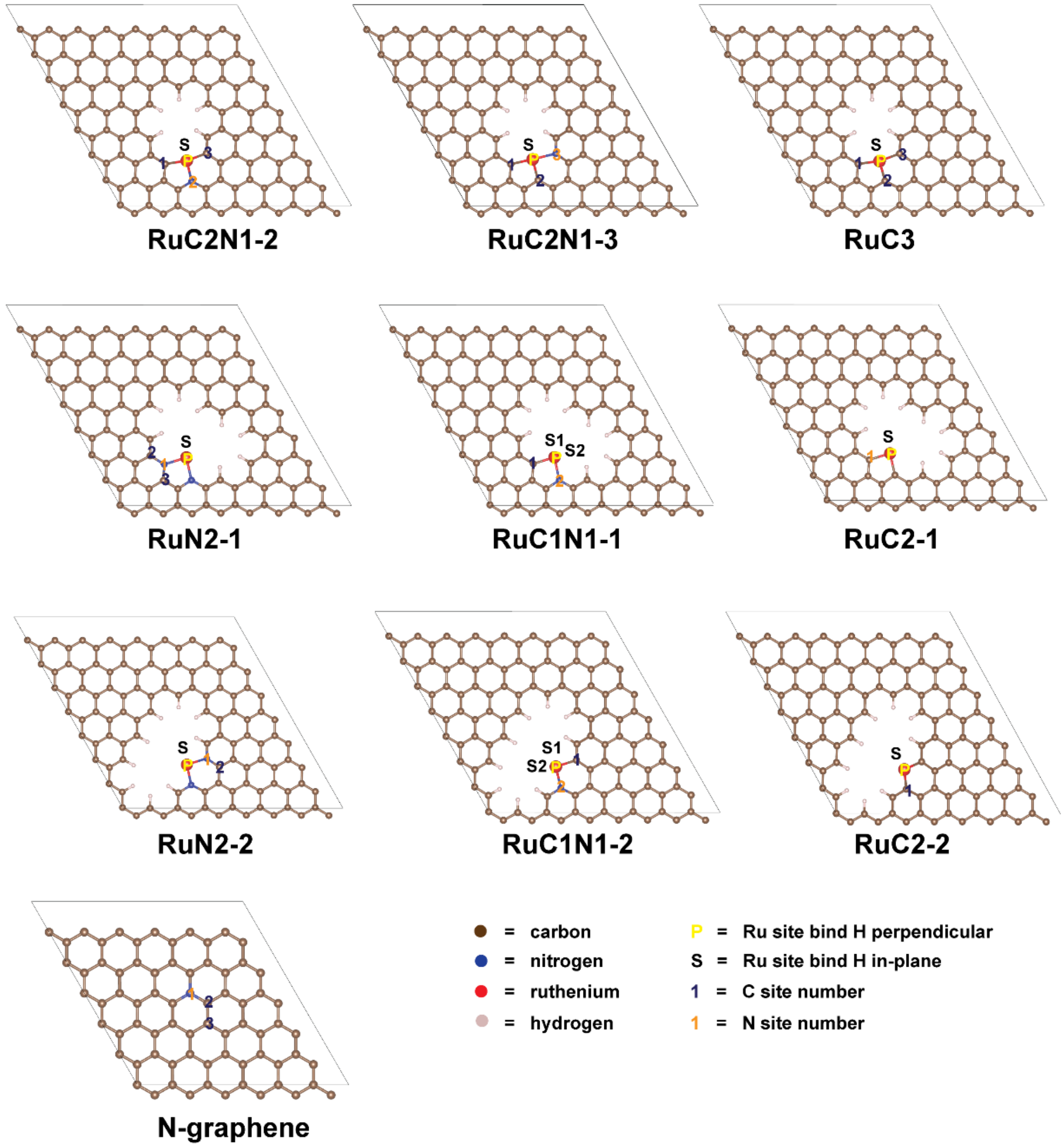


**Supplementary Figure 15 Computational models of Ru-N/Ru-C moieties.**

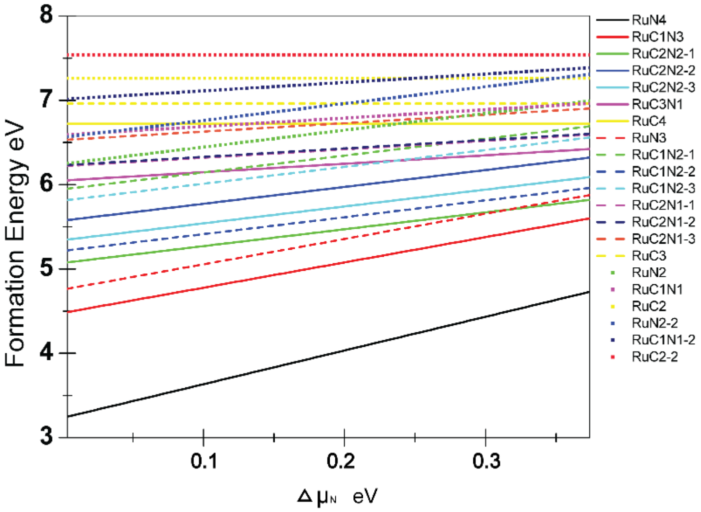


**Supplementary Figure 16 Formation energies of different Ru-N/Ru-C moieties as a function of N elemental chemical potentials**. The range of N chemical potentials is from N in $N_2$ (N rich) to N in melamine (N poor).

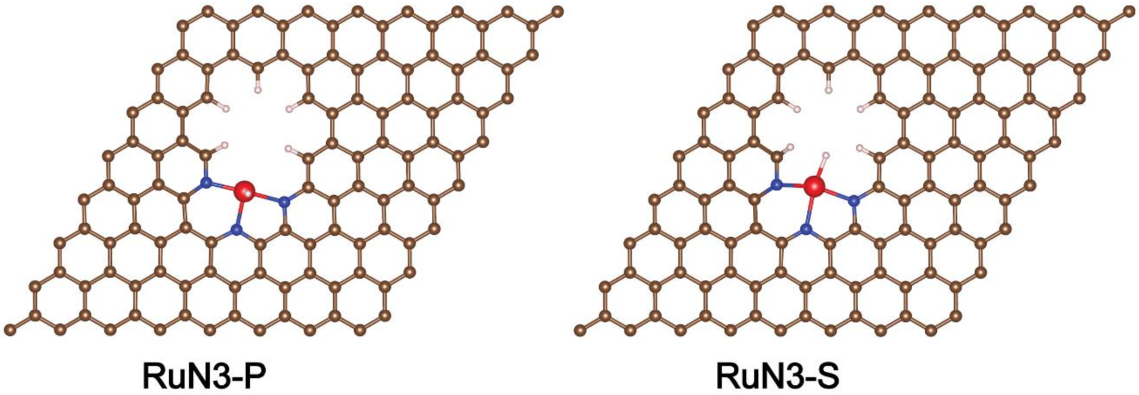


**Supplementary Figure 17 Optimized geometric structures of RuN3-P and RuN3-S.** RuN3-P and RuN3-S refer to H binding to Ru in a perpendicular and in-plane configuration, respectively.

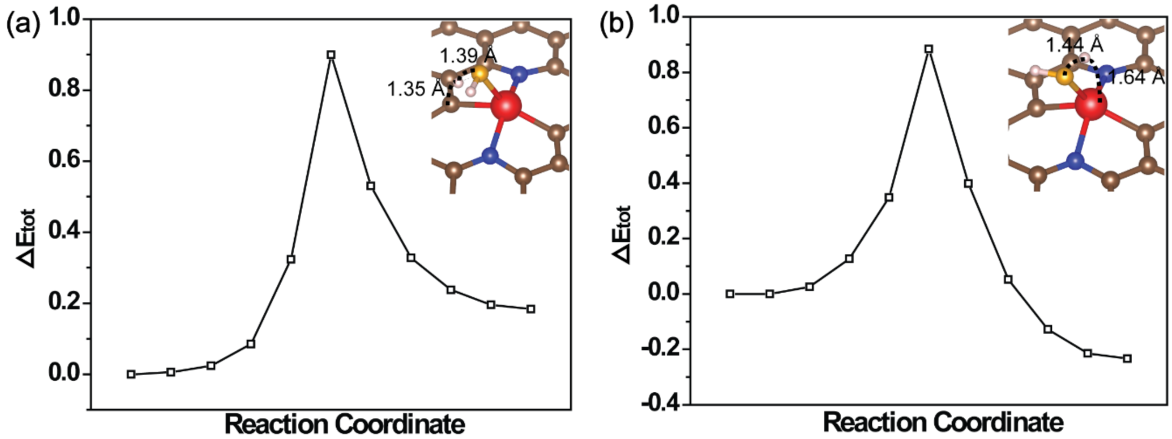


**Supplementary Figure 18 Water dissociation barrier** for (a) reaction pathway-1 and (b) reaction pathway-2. The insets are the structure of the corresponding transition state. The colors of elements are: brown for C, blue for N, red for Ru, yellow for O and white for H.

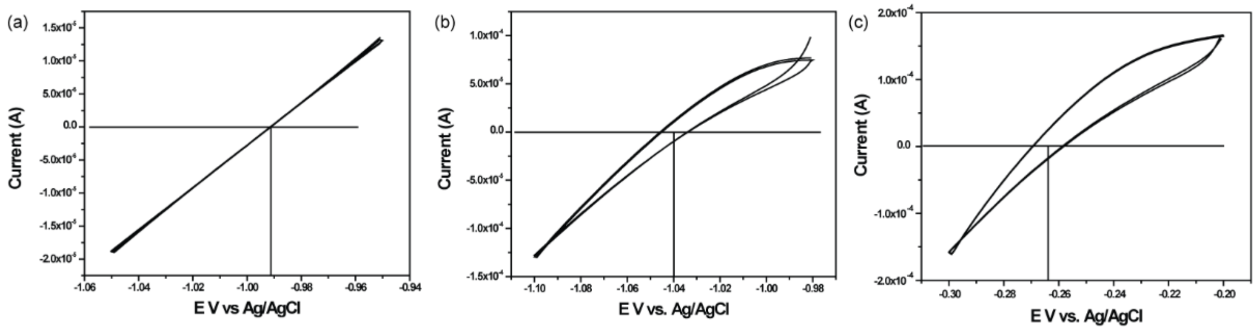


**Supplementary Figure 19 Calibration of Ag/AgCl reference electrode** in (a) 0.1 M KOH, (b) 1 M KOH, and (c) 0.5 M $H_2SO_4$. Potential scan rate 1 mV/s.

**Supplementary References**


1 Mahmood, J. *et al.* An efficient and pH-universal ruthenium-based catalyst for the hydrogen evolution reaction. *Nat. Nanotechnol.* **12**, 441 (2017).

2 Wang, P. *et al.* Precise tuning in platinum-nickel/nickel sulfide interface nanowires for synergistic hydrogen evolution catalysis. *Nat. Commun.* **8**, 14580 (2017).

3 Cao, Z. *et al.* Platinum-nickel alloy excavated nano-multipods with hexagonal close-packed structure and superior activity towards hydrogen evolution reaction. *Nat. Commun.* **8**, 15131 (2017).

4 Liu, Y. *et al.* Ru Modulation Effects in the Synthesis of Unique Rod-like Ni@Ni2P-Ru Heterostructures and Their Remarkable Electrocatalytic Hydrogen Evolution Performance. *J. Am. Chem. Soc.* **140**, 2731-2734 (2018).

5 Zheng, Y. *et al.* High Electrocatalytic Hydrogen Evolution Activity of an Anomalous Ruthenium Catalyst. *J. Am. Chem. Soc.* **138**, 16174-16181 (2016).

6 Pu, Z., Amiinu, I. S., Kou, Z., Li, W. & Mu, S. RuP2-Based Catalysts with Platinum-like Activity and Higher Durability for the Hydrogen Evolution Reaction at All pH Values. *Angew. Chem. Int. Ed.* **56**, 11559-11564 (2017).

7 Jiang, P. *et al.* Tuning the Activity of Carbon for Electrocatalytic Hydrogen Evolution via an Iridium-Cobalt Alloy Core Encapsulated in Nitrogen-Doped Carbon Cages. *Adv. Mater.* **30**, 1705324 (2018).

8 Su, J. *et al.* Ruthenium-cobalt nanoalloys encapsulated in nitrogen-doped graphene as active electrocatalysts for producing hydrogen in alkaline media. *Nat. Commun.* **8**, 14969 (2017).

9 Zhang, J. *et al.* Ruthenium/nitrogen-doped carbon as an electrocatalyst for efficient hydrogen evolution in alkaline solution. *J. Mater. Chem. A* **5**, 25314-25318 (2017).

10 Peng, Y. *et al.* Hydrogen evolution reaction catalyzed by ruthenium ion-complexed graphitic carbon nitride nanosheets. *J. Mater. Chem. A* **5**, 19499-19499 (2017).

11 Lu, B. *et al.* Nitrogen and Iron-Codoped Carbon Hollow Nanotubules as High-Performance Catalysts toward Oxygen Reduction Reaction: A Combined Experimental and Theoretical Study. *Chem. Mater.* **29**, 5617-5628 (2017).

12 Zheng, F., Yang, Y. & Chen, Q. High lithium anodic performance of highly nitrogen-doped porous carbon prepared from a metal-organic framework. *Nat. Commun.* **5**, 5261 (2014).

13 Zhang, J. *et al.* N, P-Codoped Carbon Networks as Efficient Metal-free Bifunctional Catalysts for Oxygen Reduction and Hydrogen Evolution Reactions. *Angew. Chem. Int. Ed.* **55**, 2230-2234 (2016).

14 Ruili, L., Dongqing, W., Xinliang, F. & Klaus, M. Nitrogen-Doped Ordered Mesoporous Graphitic Arrays with High Electrocatalytic Activity for Oxygen Reduction. *Angew. Chem. Int. Ed.* **49**, 2565-2569 (2010).

15 Dai, L., Xue, Y., Qu, L., Choi, H.-J. & Baek, J.-B. Metal-free catalysts for oxygen reduction reaction. *Chem. Rev.* **115**, 4823-4892 (2015).

16 Zhang, J. *et al.* Ruthenium/nitrogen-doped carbon as an electrocatalyst for efficient hydrogen evolution in alkaline solution. *J. Mater. Chem. A* **5**, 25314-25318 (2017).

17 Choi, W. I., Wood, B. C., Schwegler, E. & Ogitsu, T. Combinatorial Search for High-Activity Hydrogen Catalysts Based on Transition-Metal-Embedded Graphitic Carbons. *Adv. Energy Mater.* **5**, 1501423 (2015).

18 Li, Y. *et al.* Crystallinity Dependence of Ruthenium Nanocatalyst toward Hydrogen Evolution Reaction. *ACS Catal.*, **8**, 5714-5720 (2018).
