# Supplementary material for: Ruthenium atomically dispersed in carbon outperforms platinum toward hydrogen evolution in alkaline media
Source: Nat Commun. 2019 Feb 7;10:631. doi: 10.1038/s41467-019-08419-3 (PMC6367462; doi:10.1038/s41467-019-08419-3)
Supplement: Supplementary file 2 — Description of Additional Supplementary Files [file 41467_2019_8419_MOESM2_ESM.pdf]

Description of Additional Supplementary Files

File Name: Supplementary Movie 1
Description: **Water dissociation dynamics.** Hydrogen binds to the carbon adjacent to ruthenium.

File Name: Supplementary Movie 2
Description: **Water dissociation dynamics.** Hydrogen binds to ruthenium.
